# WOMAC-based recovery after total knee arthroplasty in a multiethnic Asian cohort: a 5-year registry-based study of interval-specific predictors of improvement

**DOI:** 10.1007/s11136-026-04276-y

**Published:** 2026-05-03

**Authors:** Eau Liang Teow, Xin Zhang, Wei Loong Barry Tan, Chee Hoe Kong, Harish Sivasubramanian, Xun Li, Melvin Kian Loong Tan, Zi Qiang Glen Liau, Nick Bansback, Nan Luo, Wai San Wilson Tam, Ling Jie Cheng

**Affiliations:** 1 Alice Lee Centre for Nursing Studies, Yong Loo Lin School of Medicine, National University of Singapore, Singapore, Singapore; 2 Department of Nursing, Ng Teng Fong General Hospital, Singapore, Singapore; 3 Saw Swee Hock School of Public Health, National University of Singapore, Singapore, Singapore; 4 Division of Adult Reconstruction and Joint Replacement Surgery, Department of Orthopaedic Surgery, National University Hospital, Singapore, Singapore; 5 Department of Orthopaedic Surgery, Yong Loo Lin School of Medicine, National University of Singapore, Singapore, Singapore; 6 Division of Hip and Knee Surgery, Department of Orthopaedic Surgery, Ng Teng Fong General Hospital, Singapore, Singapore; 7 School of Engineering, Computing, and Mathematics, Oxford Brookes University, Oxford, UK; 8 Adult Reconstruction & Joint Replacement Service, Orthopaedic Surgery, Alexandra Hospital, Singapore, Singapore; 9 School of Population and Public Health, University of British Columbia, Vancouver, BC, Canada; 10 National Perinatal Epidemiology Unit, Nuffield Department of Women’s and Reproductive Health, University of Oxford, Oxford, UK

**Keywords:** Patient-reported outcomes, Total knee arthroplasty, Knee osteoarthritis, Longitudinal cohort, Surgery registry

## Abstract

**Background:**

Patient-reported outcomes (PROs) after total knee arthroplasty (TKA) are well-documented in Western populations, but long-term recovery and its determinants in multiethnic Asian populations remain poorly understood. We aimed to characterise 5-year WOMAC recovery after TKA and to identify patient, clinical, and surgical factors associated with the odds of improvement at each consecutive postoperative interval in a multiethnic Asian cohort.

**Methods:**

This registry-based cohort study used prospectively collected data from a tertiary hospital in Singapore. We included 4964 consecutive cases with osteoarthritis undergoing primary TKA between December 1, 2008, and December 31, 2023. The primary outcomes were changes in the Western Ontario and McMaster Universities Osteoarthritis Index (WOMAC) total and subscale scores, measured at baseline and at 6 months, 1, 2, and 5 years postoperatively.

**Results:**

The mean (SD) total WOMAC score improved from 38.6 (15.1) at baseline to 7.5 (9.2) at 5 years. The greatest improvement occurred within the first 6 months (mean change, 27.6 points; P value < .001). In multivariable interval-specific analyses, older age (≥ 75 years; OR 0.55, 95% CI 0.39–0.79) and the presence of one or more comorbidities (OR 0.83, 95% CI 0.71–0.96) were independently associated with lower odds of long-term improvement. Interval-specific associations with ethnicity were also observed for pain and stiffness.

**Conclusions:**

In this large, multi-ethnic Asian cohort, TKA was associated with substantial and durable WOMAC improvements, primarily within the first 6 months. Interval-specific predictors of improvement were dominated by patient-level factors, notably age, comorbidity, and ethnicity, while surgical variables showed limited association. These findings support the potential value of patient-centred risk stratification and culturally responsive perioperative care in optimising long-term outcomes.

**Supplementary Information:**

The online version contains supplementary material available at 10.1007/s11136-026-04276-y.

## Introduction

Osteoarthritis is a progressive musculoskeletal disorder leading to pain, impaired mobility, and functional limitation, and it remains a leading cause of disability worldwide [1]. Its prevalence is rising with population ageing and increasing obesity, affecting over 370 million people globally in 2021 and projected to impact more than 640 million by 2050 [2]. In response to this growing burden, total knee arthroplasty (TKA) has become one of the most effective and frequently performed surgical interventions for restoring quality of life in end-stage knee osteoarthritis [3].

Although TKA typically provides substantial pain relief and improved mobility, up to 20% of patients remain dissatisfied, often due to persistent pain or limited functional gain [4]. A significant limitation in understanding these outcomes is that the vast majority of long-term data arise from Western registries, such as the Australian Orthopaedic Association National Joint Replacement Registry [5] and the United Kingdom (National Joint Registry of England and Wales) [6], which represent ethnically homogeneous populations. These data may not account for the cultural, linguistic, and lifestyle differences that influence recovery patterns in Asian cohorts. While patient-reported outcomes (PROs) have emerged as critical tools for evaluating TKA effectiveness and facilitating shared decision-making [7], most studies report on short-term follow-up of 6 months to 2 years [8–13] and predate major advances in surgical technique and perioperative care [8, 9, 14].

The evidence base from Asia is currently confined to smaller cohorts with limited follow-up, and multiethnic Asian populations are persistently underrepresented in the literature [4, 15]. This is a critical gap, as cultural context, health beliefs, and activity patterns are recognised as important influences on recovery and satisfaction [15, 16]. While several studies have identified clinical and sociodemographic predictors of PROs after TKA [17], including gender [8, 9], age [9–11, 13], body mass index (BMI) [9, 18, 19], presence of comorbidities [9, 11, 20, 21], baseline severity [11, 13], and perioperative surgical-related factors [22], these factors have predominantly been evaluated in the short term. Detailed analyses of ethnicity-specific recovery patterns over longer periods are lacking, leaving the durability and transferability of earlier findings to contemporary, diverse populations uncertain. Singapore's multicultural population presents a unique opportunity to investigate how ethnic background may influence functional recovery and pain perception patterns following TKA.

Therefore, this study aimed to (1) describe mean Western Ontario and McMaster Universities Osteoarthritis index (WOMAC) total and subscale scores at baseline (T0), 6 months (T1), 1 year (T2), 2 years (T3), and 5 years (T4) post-surgery; (2) quantify the magnitude and timing of mean change in pain, stiffness, and physical function across each interval; and (3) identify sociodemographic, clinical, and surgical factors independently associated with the odds of WOMAC improvement at each of four consecutive postoperative intervals within Singapore’s multiethnic TKA population.

## Methods

### Ethical approval and registration

The study was conducted in accordance with the Declaration of Helsinki and received approval from the Domain Specific Review Board, National Healthcare Group, Singapore (DSRB 2024/00191). All registry data were de-identified and anonymised prior to analysis.

### Reporting standards

We reported the study following the Strengthening the reporting of cohort studies in surgery (STROCSS), REporting of Studies Conducted Using Observational Routinely-Collected Health Data (RECORD) and Strengthening the Reporting of Observational Studies in Epidemiology (STROBE) statement (Table S1) [23, 24].

### Study design and setting

This retrospective analysis used prospectively collected data from the National University Hospital arthroplasty registry (TKA; operational from 2009; data extracted in 2024), one of the largest local TKA databases at a tertiary referral hospital in Singapore. The registry forms part of an institutional initiative to monitor surgical outcomes and support research within Asian populations. Cases undergoing primary TKA from December 2008 were entered retrospectively at registry inception; accordingly, the analytic inclusion period (December 1, 2008, to December 31, 2023) precedes the registry’s operational start date.

### Data collection

Data were collected at scheduled timepoints: baseline (T₀), 6 months (T₁), 1 year (T₂), 2 years (T₃), and 5 years (T₄) postoperatively. Informed consent was obtained from all participants at registry enrolment. While the institutional registry collected various PRO measures (PROMs) over its history, this analysis was restricted to cases with valid WOMAC data (defined as ≥ 13 of 24 items answered) at baseline, using the WOMAC as the sole patient-reported outcome measure for this longitudinal analysis.

PROMs were collected via interviewer-administered questionnaires during clinic visits or by telephone interviews as needed. Questionnaires were provided in English and Mandarin [25]. Sociodemographic information was recorded at registry enrolment. Ethnicity was classified according to Singapore’s official categorisation into Chinese, Malay, Indian, and Others, the latter comprising Eurasian, Caucasian, and other minority groups not otherwise specified. Singapore is a multiethnic city-state in Southeast Asia where these groups have coexisted since independence in 1965, with distinct cultural, linguistic, and health-seeking characteristics.

Comorbidities were extracted from electronic medical records and included documented diagnoses of diabetes mellitus, hypertension, hyperlipidaemia, ischaemic heart disease, cardiac failure, cerebrovascular disease, chronic lung disease, or chronic kidney disease. The presence of one or more comorbid conditions was coded as a binary variable (any vs. none). Additional clinical variables, including intraoperative details (implant model, surgical duration, blood loss) and postoperative outcomes, were extracted by trained research coordinators using standardised data collection forms. Regular data quality audits were conducted to ensure accuracy and completeness. All data were managed using Research Electronic Data Capture (REDCap) [26, 27].

### Sampling and eligibility criteria

Cases were eligible if they had a clinical diagnosis of osteoarthritis, underwent primary TKA between December 1, 2008, and December 31, 2023, and completed baseline PROMs with at least 13 of 24 valid responses for the WOMAC items.

### Dependent variable

The primary outcome was the change in patient-reported pain, stiffness, and function, as measured by the WOMAC instrument [28]. The WOMAC instrument consists of 24 items across three domains: pain (5 items), stiffness (2 items), and physical function (17 items), each scored on a Likert scale from 0 (none) to 4 (extreme), with higher scores indicating greater impairment [28]. Total WOMAC scores were standardised to a 0–100 scale (lower values = better health), and subscale means reported on the original 0–4 scale (higher values = more severe symptoms).

The WOMAC was selected for its demonstrated sensitivity to change following TKA, its comprehensive historical collection within the institutional registry, and its established validity in the Singaporean context [28–30]. Change in WOMAC scores was calculated between consecutive follow-up timepoints (T_0_–T_1_, T_1_–T_2_, T_2_–T_3_, and T_3_–T_4_). This continuous change score was then dichotomised to represent improvement (a positive change) versus no improvement or worsening (a zero or negative change) to facilitate clinical interpretation. A minimum clinically important difference (MCID) threshold was not imposed. This decision was based on the absence of validated MCID cut-offs for multi-ethnic Asian populations and the substantial inter-study variability in reported thresholds (9–15 points) [31, 32]. Consequently, using the direction of change represents a consistent and defensible approach for diverse populations where ethnic-specific MCID values remain undetermined.

### Independent variables

Independent variables included sociodemographic factors (age, sex, ethnicity) and clinical characteristics such as BMI, medical history, and perioperative details. Surgical variables (implant model, surgical duration, blood loss, tourniquet time, laterality, thromboembolic prophylaxis regimen) were selected based on their established associations with postoperative pain, recovery, and functional outcomes in the arthroplasty literature. Variables were grouped into clinically relevant categories for analysis (see Table S2 for details).

### Statistical methods

Descriptive statistics were used to summarise patient characteristics; continuous variables were reported as means and standard deviations (SDs) or medians and interquartile ranges (IQRs), while categorical variables were presented as counts and percentages. WOMAC scores were analysed by total and domain at each timepoint (T_1_–T_4_).

To identify factors associated with interval-specific improvement, we first performed univariable analyses, in which each candidate predictor was examined separately against the binary outcome (improvement vs. no improvement) at each interval. Variables with a P value < .10 in these univariable tests were then included in multivariable mixed-effects logistic regression models fitted separately at each interval. These interval-specific models, which estimated the odds of improvement on the WOMAC total and subscale scores, treated the surgeon as a random effect to account for clustering and were adjusted for the year of surgery and the baseline WOMAC score. We also examined potential effect modification by including interaction terms in the models. Interactions were evaluated at each interval using Wald tests and retained in the final models if statistically significant. Separately, model assumptions (collinearity, specification) were assessed via variance inflation factors (< 10) and the Pregibon link test (*Hat*^*2*^); a non-significant result indicated appropriate specification. Odds ratios (ORs) and 95% confidence intervals (CIs) were reported.

Missing items within a single WOMAC questionnaire were addressed using the half-rule: if 13 or more of the 24 items were answered, a score was calculated by imputing the mean of the observed responses; otherwise, the score for that timepoint was treated as missing [33]. For longitudinal analyses between two timepoints, cases were included only if they had valid WOMAC data (< 13 missing items) for both intervals. A sensitivity analysis was performed using multiple imputation by chained equations (MICE) to assess the potential impact of patient attrition under a missing-at-random assumption [34]. Furthermore, to investigate potential non-response bias, the baseline characteristics of cases with incomplete data were compared with those included in the final analysis.

All analyses were conducted using Stata version 18.0 (StataCorp). No adjustment was made for multiple comparisons, consistent with the exploratory nature of the analysis.

## Results

### Participant flow and data completeness

**Fig. 1:**
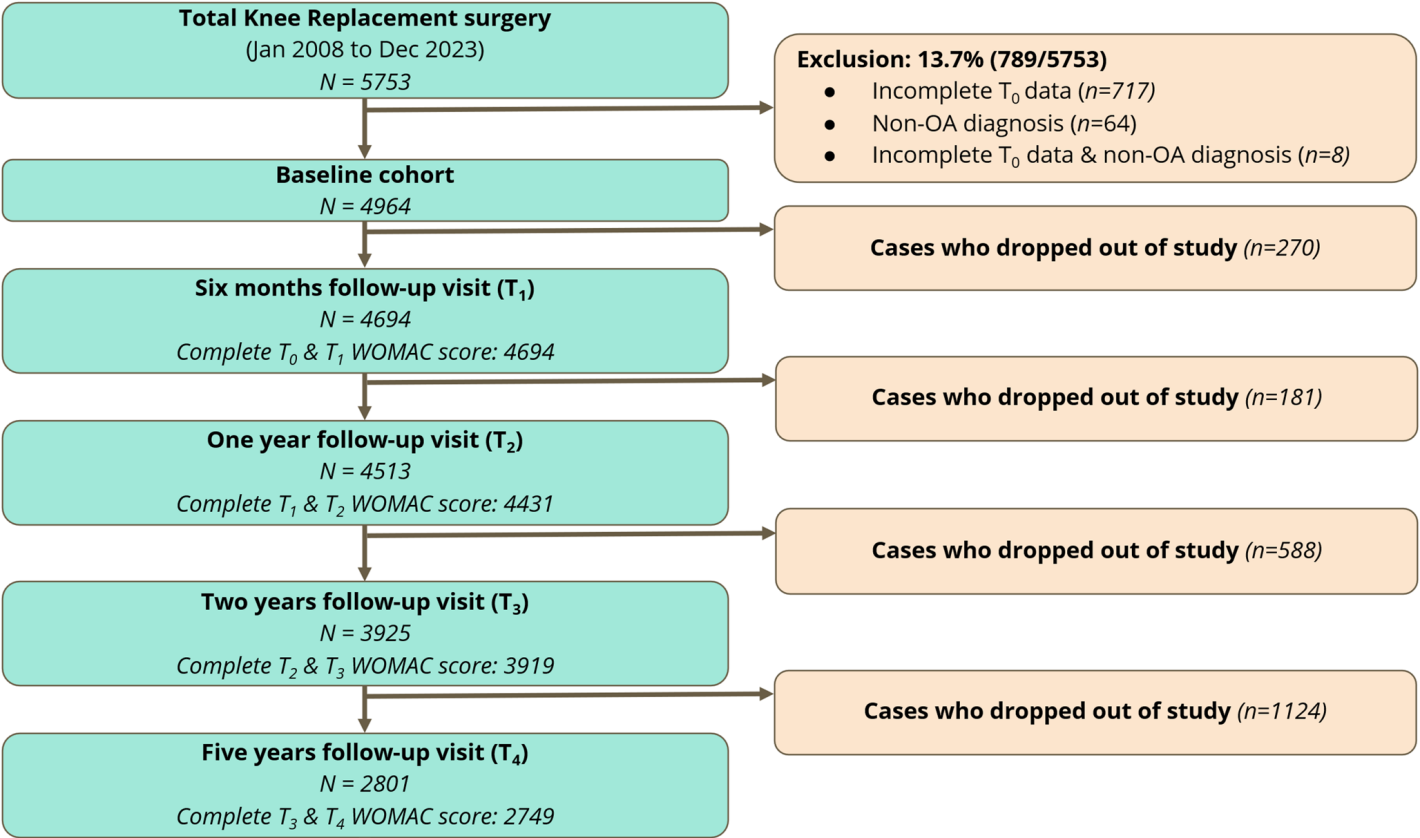
Flowchart of cases inclusion and follow-up for total knee replacement cohort, with exclusion and dropout numbers at each longitudinal timepoint. (T0: Pre-op (baseline). T1: 6 months follow-up. T2: 1-year follow-up. T3: 2-year follow-up. T4: 5-year follow-up; OA: Osteoarthritis; WOMAC: Western Ontario and McMaster Universities Osteoarthritis Index)

Of the 5753 cases assessed for eligibility, 789 (13.7%) were excluded (primarily due to incomplete baseline data; n = 717), yielding a final analytical cohort of 4964 cases (Fig. 1). The number of cases available for follow-up was 4694 at 6 months, 4513 at 1 year, 3925 at 2 years, and 2801 at 5 years, which represents a 56.4% follow-up rate at the 5-year timepoint. Among cases who were followed up, the completeness of WOMAC data remained high throughout the study period, ranging from 98.1 to 100% at the various intervals.

### Participant characteristics

At baseline, the mean (SD) age of the cohort was 67.0 (8.1) years, with 44.0% of cases aged 65–74 years and 18.0% aged 75 years or older. The cohort was predominantly female (3450 cases [69.5%]) and of Chinese ethnicity (3519 cases [70.9%]). The mean (SD) BMI was 28.0 (4.9), and comorbidities were present in 3841 cases (77.3%). Regarding surgical details, most procedures were unilateral (96.3%), 60.6% had a surgical duration exceeding 100 min, and 66.5% had a tourniquet time of under 100 min. The detailed distribution of sociodemographic and clinical characteristics is presented in Table 1.

**Table 1:** Distribution of sociodemographic and clinical variables across the cohorts

Variables	T_0_ (N = 4964)	T_0_–T_1_ (N = 4694)	T_1_–T_2_ (N = 4431)	T_2_–T_3_ (N = 3919)	T_3_–T_4_ (N = 2749)
	N (%)	N (%)	N (%)	N (%)	N (%)
Age, years (Mean [SD])	67.0 [8.09]	67.0 [8.07]	67.0 [8.00]	66.7 [7.99]	66.1 [8.05]
Young adult (< 55)	316 (6.4)	301 (6.4)	278 (6.3)	266 (6.8)	215 (7.8)
Older adult (55–64)	1571 (31.6)	1477 (31.5)	1389 (31.3)	1250 (31.9)	941 (34.2)
Early elder (65–74)	2183 (44.0)	2077 (44.2)	1981 (44.7)	1754 (44.8)	1174 (42.7)
Late elder (≥ 75)	894 (18.0)	839 (17.9)	783 (17.7)	649 (16.6)	419 (15.2)
Gender					
Female	3448 (69.5)	3264 (69.5)	3079 (69.5)	2740 (69.9)	1961 (71.3)
Male	1516 (30.5)	1430 (30.5)	1352 (30.5)	1179 (30.1)	788 (28.7)
Ethnicity					
Chinese	3519 (70.9)	3361 (71.6)	3203 (72.3)	2832 (72.3)	2015 (73.3)
Malay	749 (15.1)	705 (15.0)	660 (14.9)	590 (15.1)	387 (14.1)
Indian	595 (12.0)	559 (11.9)	514 (11.6)	451 (11.5)	325 (11.8)
Others	101 (2.0)	69 (1.5)	54 (1.2)	46 (1.2)	22 (0.8)
BMI, kg/m^2^ (Mean [SD])	28.0 [4.87]	27.9 [4.82]	27.9 [4.81]	27.9 [4.82]	27.9 [4.76]
Underweight (< 18.5)	40 (0.8)	37 (0.8)	34 (0.8)	29 (0.7)	22 (0.8)
Normal (18.5–24.9)	1405 (28.3)	1332 (28.4)	1259 (28.4)	1118 (28.5)	791 (28.8)
Overweight (25–29.9)	2054 (41.4)	1959 (41.7)	1855 (41.9)	1634 (41.7)	1137 (41.4)
Obese (≥ 30)	1465 (29.5)	1366 (29.1)	1283 (29.0)	1138 (29.0)	799 (29.1)
Presence of ≥ 1 comorbidity					
Yes	3835 (77.3)	3635 (77.4)	3443 (77.7)	3018 (77.0)	2102 (76.5)
No	1129 (22.7)	1059 (22.6)	988 (22.3)	901 (23.0)	647 (23.5)
Type of TKA procedure					
Bilateral TKA	183 (3.7)	159 (3.4)	145 (3.3)	127 (3.2)	79 (2.9)
Unilateral and staged TKA	4780 (96.3)	4534 (96.6)	4285 (96.7)	3791 (96.8)	2669 (97.1)
Surgical time, mins					
≤ 100	1957 (39.4)	1855 (39.5)	1753 (39.6)	1518 (38.8)	1077 (39.2)
> 100	3005 (60.6)	2837 (60.5)	2676 (60.4)	2399 (61.2)	1671 (60.8)
Tourniquet time, mins					
No tourniquet	46 (0.9)	46 (1.0)	44 (1.0)	40 (1.0)	30 (1.1)
≤ 100	3252 (66.5)	3061 (66.1)	2890 (66.1)	2532 (65.2)	1755 (64.4)
> 100	1593 (32.6)	1523 (32.9)	1438 (32.9)	1311 (33.8)	942 (34.5)
Type of implant model					
Model J&J	969 (19.5)	920 (19.6)	870 (19.6)	791 (20.2)	573 (20.8)
Model S&N	1216 (24.5)	1134 (24.2)	1082 (24.4)	887 (22.7)	468 (17.0)
Models S, B & Others	857 (17.3)	810 (17.3)	767 (17.3)	714 (18.2)	606 (22.1)
Model Z	1920 (38.7)	1828 (39.0)	1710 (38.6)	1525 (38.9)	1102 (40.1)
Type of anesthesia					
GA	3961 (79.8)	3752 (80.0)	3535 (79.8)	3191 (81.5)	2377 (86.5)
Non-GA	1000 (20.2)	939 (20.0)	893 (20.2)	725 (18.5)	372 (13.5)
Blood loss, ml (s)					
< 250	4159 (88.8)	3937 (88.8)	3712 (88.7)	3465 (89.0)	2408 (87.7)
≥ 250	523 (11.2)	494 (11.2)	474 (11.3)	430 (11.0)	338 (12.3)
Thromboembolic prophylaxis					
Pharm & Mech	2923 (59.0)	2757 (58.8)	2639 (59.6)	2253 (57.5)	1334 (48.5)
Pharm or Mech	1969 (39.7)	1868 (39.8)	1729 (39.1)	1611 (41.1)	1366 (49.7)
None	66 (1.3)	63 (1.3)	57 (1.3)	53 (1.4)	49 (1.8)
Post-op complication					
No complication	3919 (84.4)	3707 (84.4)	3507 (84.4)	3265 (84.3)	2284 (83.8)
Complication	722 (15.6)	687 (15.6)	647 (15.6)	608 (15.7)	441 (16.2)
Length of stay, days					
≤ 5	3687 (74.5)	3484 (74.5)	3292 (74.5)	2867 (73.4)	1886 (68.8)
> 5	1261 (25.5)	1195 (25.5)	1125 (25.5)	1039 (26.6)	856 (31.2)

T_0_: Pre-op (baseline). T_1_: 6 months follow-up. T_2_: 1 year follow-up. T_3_: 2-year follow-up. T_4_: 5-year follow-up; Others (Ethnicity): Minority populations that are not part of Singapore’s three main ethnic groups (Chinese, Malay, and Indian); Pharm: Pharmacological thromboembolic prophylaxis that includes use of Low-Molecular-Weight-Heparins (LMWHs), heparins, and aspirin to prevent post-operative Venous thromboembolism (VTE); Mech: Mechanical thromboembolic prophylaxis that includes compression devices such as thromboembolic deterrent stockings (TEDs), calf pumps and foot pumps to prevent post-operative VTE

### Mean WOMAC total and subscale scores over time

Substantial improvement in WOMAC total scores was observed, declining from a mean of 38.6 (SD = 15.1) at baseline to 7.5 (SD = 9.2) at 5 years. The most pronounced improvement occurred within the first 6 months (mean change, − 27.6 points; *P* < .001), after which mean improvement plateaued, with negligible mean change beyond 1 year.

**Fig. 2:**
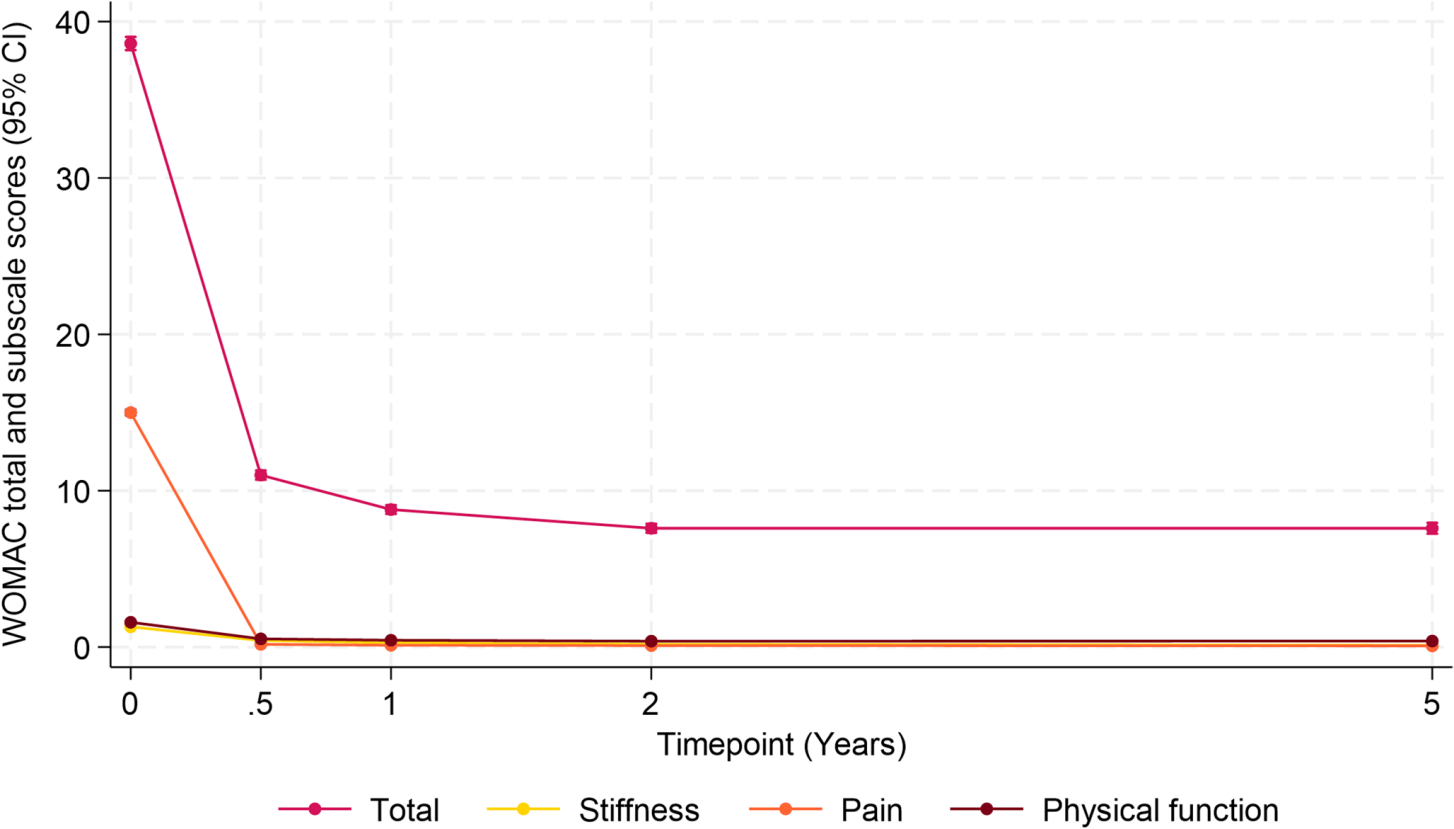
Mean WOMAC total and subscale (stiffness, pain, physical function) scores with 95% confidence intervals at baseline, 6 months, 1 year, 2 years, and 5 years post-surgery. This figure is a descriptive summary of mean WOMAC scores at scheduled postoperative timepoints and is not a model-based trajectory

This pattern was consistent across all subscales. Pain showed the largest improvement, with the mean (SD) score decreasing from 1.50 (0.67) to 0.08 (0.24). Stiffness scores declined from 1.30 (1.02) to 0.12 (0.39), and physical function scores improved from 1.59 (0.65) to 0.39 (0.47) over the 5-year period (Fig. 2 and Table 2).

**Table 2:** Descriptive statistics and paired t-tests of WOMAC total and subscale scores at each timepoint

Outcome	Timepoint	N	Improvement from the preceding time point, n (%)	Mean (SD)	Mean change from the preceding timepoint (95% CI)	*p* value
Total	Baseline	4964		38.6 (15.1)	–	–
	6 months	4694	4503 (95.9)	11.0 (10.2)	27.6 (27.1, 28.0)	< 0.001
	1 year	4431	2454 (55.4)	8.8 (9.4)	2.17 (1.88, 2.46)	< 0.001
	2 years	3919	2147 (54.8)	7.6 (9.0)	1.38 (1.08, 1.68)	< 0.001
	5 years	2749	1366 (49.7)	7.6 (9.2)	0.25 (-0.12, 0.63)	0.182
Stiffness	Baseline	4961		1.30 (1.02)	–	–
	6 months	4692	2956 (63.0)	0.42 (0.64)	0.87 (0.84, 0.91)	< 0.001
	1 year	4430	1219 (27.5)	0.28 (0.54)	0.14 (0.12, 0.16)	< 0.001
	2 years	3918	857 (21.9)	0.19 (0.47)	0.09 (0.07, 0.11)	< 0.001
	5 years	2749	461 (16.8)	0.12 (0.39)	0.08 (0.06, 0.10)	0.182
Pain	Baseline	4955		1.50 (0.67)	–	–
	6 months	4682	4490 (95.7)	0.18 (0.34)	1.32 (1.30, 1.34)	< 0.001
	1 year	4427	1167 (26.3)	0.12 (0.29)	0.06 (0.05, 0.07)	< 0.001
	2 years	3916	743 (19.0)	0.10 (0.26)	0.02 (0.01, 0.03)	< 0.001
	5 years	2748	418 (15.2)	0.08 (0.24)	0.02 (0.01, 0.03)	< 0.001
Physical function	Baseline	4964		1.59 (0.65)	–	–
	6 months	4694	4411 (94.0)	0.53 (0.49)	1.06 (1.04, 1.09)	< 0.001
	1 year	4431	2368 (53.4)	0.44 (0.46)	0.09 (0.07, 0.10)	< 0.001
	2 years	3919	2097 (53.5)	0.38 (0.45)	0.06 (0.05, 0.08)	< 0.001
	5 years	2749	1391 (50.6)	0.39 (0.47)	0.004 (-0.02, 0.02)	0.687

*CI* confidence interval, *SD* standard deviation, *WOMAC* Western Ontario and McMaster Universities Osteoarthritis Index, *N* total number of participants assessed at each timepoint, *n* number of participants showing improvement, *%* proportion of participants showing improvement, *p* values: derived from paired *t*-tests comparing each follow-up with the preceding timepoint. This table presents a descriptive summary of mean WOMAC change between scheduled postoperative timepoints; it is not a model-based trajectory analysis

### Factors associated with improvement in WOMAC scores

In univariable analyses (Table S3), variables meeting the threshold were entered into multivariable models adjusting for baseline WOMAC score, surgery year, and surgeon random effects.

**Fig. 3:**
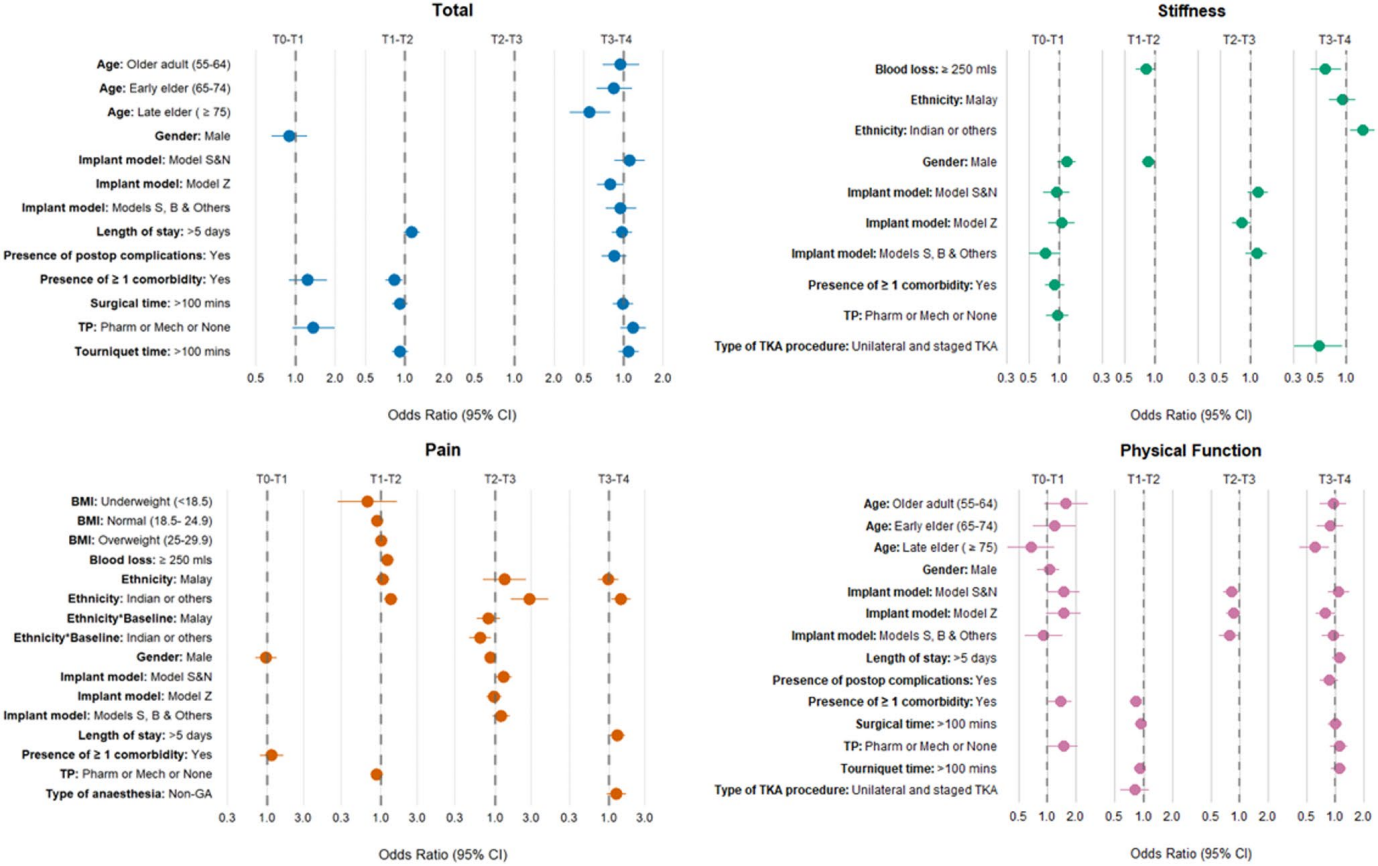
Forest plots of adjusted odds ratios (ORs) with 95% confidence intervals (CIs) from interval-specific multivariable logistic regression models for WOMAC total and subscale (stiffness, pain, physical function) improvement at each of four consecutive postoperative intervals (T0–T1, T1–T2, T2–T3, T3–T4). Estimates should be interpreted within each interval; the covariate set was determined by interval-specific screening and is not harmonised across intervals. Cross-interval comparison of effect magnitudes is not supported

In the multivariable analyses, no sociodemographic or clinical variable was independently associated with improvement during the early postoperative period (T₀–T₁). In later periods, two factors were associated with lower odds of improvement. Between 6 months and 1 year, the presence of one or more comorbidities was associated with reduced odds of improvement (OR 0.83; 95% CI 0.71–0.96). In the long term (between 2 and 5 years), age ≥ 75 years was associated with a similar reduction in the odds of improvement (OR 0.55; 95% CI 0.39–0.79). Sex, surgical variables, and postoperative course showed no clear association with improvement at any interval (Table S3). Interval-specific multivariable results are summarised in Fig. 3 and should be interpreted within each interval; the figure is not intended for cross-interval comparison.

### Stiffness subscale

In the multivariable models for the stiffness subscale, only a few factors were associated with outcomes, primarily in the long term. Between 2 and 5 years postoperatively, cases of Indian or other ethnicity had greater odds of improvement compared with cases of Chinese ethnicity (OR 1.45; 95% CI 1.10–1.93). In contrast, unilateral TKA (OR 0.53; 95% CI 0.31–0.91) and intraoperative blood loss ≥ 250 mL (OR 0.62; 95% CI 0.44–0.89) were associated with lower odds of improvement during this period.

### Pain subscale

For the pain subscale, ethnicity was the only consistent predictor of long-term improvement. Compared with patients of Chinese ethnicity, those of Indian or other ethnicity had greater odds of improvement at T₁–T₂ (OR 1.35, 95% CI 1.10, 1.66), T₂–T₃ (OR 2.87, 95% CI 1.62, 5.08), and T₃–T₄ (OR 1.45, 95% CI 1.08, 1.94). A significant interaction between ethnicity and baseline pain was also found between 1 and 2 years postoperatively (*P* = .03).

### Physical function subscale

In the early recovery period (T₁–T₂), cases who received single-modality thromboembolic prophylaxis had greater odds of improvement (OR 1.46; 95% CI 1.02–2.10). Subsequently, between T₂–T₃, the presence of ≥ 1 comorbidity was associated with lower odds of improvement (OR 0.83; 95% CI 0.72–0.96). In the long term (T₃–T₄), both age ≥ 75 years (OR 0.61; 95% CI 0.43–0.87) and use of implant model Z (OR 0.79; 95% CI 0.63–1.00) were also associated with reduced odds of improvement.

### Model diagnostics and sensitivity analyses

Model fit was acceptable for all analyses, and variance inflation factors were uniformly low (mean VIF < 2.2), indicating no evidence of multicollinearity. The results were consistent between the primary complete-case analysis and the sensitivity analysis using multiple imputation (Table S4 and Table S5).

## Discussion

In this large, 5-year registry-based study of a multiethnic Asian cohort, TKA was associated with substantial and durable improvements in patient-reported pain, stiffness, and physical function. The most pronounced gains occurred within the first 6 months and were sustained long-term, a recovery pattern consistent with established findings from both Western and other Asian cohorts [4, 12, 13, 16]. However, by providing rare long-term insight into this population, our analysis reveals that these benefits are not uniform; interval-specific improvements in WOMAC scores were independently associated with patient-level factors, notably age, comorbidity burden, and ethnicity, rather than procedural variables.

A notable observation in this study was the variation in interval-specific improvement across ethnic groups. Patients of Indian and other minority ethnicities had greater odds of improvement in pain and stiffness compared with the majority Chinese group. This observation is likely multifactorial. It may partly reflect higher baseline WOMAC scores and subsequent regression to the mean [35], as well as broader contextual factors operating within Singapore’s healthcare and social environment. Differences in the timing of surgical presentation and patterns of healthcare utilisation across ethnic groups have been documented previously, which may influence both baseline severity and subsequent recovery. In addition, variation in pain perception, coping strategies, and social support structures may contribute to postoperative recovery [15]. Taken together, these contextual and psychosocial factors may help explain the observed differences in recovery patterns [36]. These findings suggest that preoperative disparities may translate into heterogeneous postoperative outcomes and underscore the importance of culturally responsive perioperative care. Further mixed-methods research is needed to clarify the relative contributions of biological, psychosocial, and structural factors and to inform equitable, targeted interventions for Singapore’s diverse populations.

A prominent observation in this cohort was the negative association between older age and a higher comorbidity burden with long-term functional recovery [11, 16]. Our analysis demonstrated that the presence of one or more comorbidities was independently associated with reduced odds of long-term improvement. This observation is consistent with international literature documenting the impact of multimorbidity on TKA response, with plausible mechanisms including diminished physiological reserve and delayed rehabilitation [37, 38]. The persistence of these associations has important clinical implications. Preoperative risk assessment and patient counselling should focus on the patient's overall health status and total comorbidity burden, rather than on isolated diagnoses. These findings also reinforce the potential value of pre-habilitation programmes, multimorbidity optimisation, and stratified postoperative pathways, particularly for older patients with limited physiological reserve.

Contrary to some reports that emphasise surgical details [17, 22, 39, 40], most operative variables such as tourniquet time, surgical duration, and implant model showed only a limited and inconsistent association with long-term outcomes in our cohort. This is broadly consistent with evidence from other large registries, which suggest that patient-level factors may play a more prominent role than implant choice or technical variables in determining patient-reported outcomes and satisfaction [16, 41, 42]. These findings suggest that future efforts to optimise TKA outcomes may benefit from greater attention to patients’ overall medical and psychosocial health alongside ongoing refinements in surgical technique.

These results have several implications for clinical practice and research. The marked improvement observed within the first 6 months highlights this period as a critical window for intervention, where postoperative resources such as intensive physiotherapy and structured pain management should be prioritised. Our findings support the use of risk stratification to inform perioperative planning; patients aged ≥ 75 years and those with multiple comorbidities warrant enhanced preoperative counselling and targeted health optimisation. Furthermore, routine interval-based monitoring of patient-reported outcomes can help identify patients with slower recovery who may benefit from additional support. This study also highlights the need for mixed-methods research to investigate the psychosocial, cultural, and support-related mechanisms that underlie the variability in outcomes, particularly in Asia's diverse patient populations. Future work could incorporate qualitative methods and digital monitoring, including wearable sensors, to better understand the behavioural and cultural factors that influence recovery. Finally, cross-regional registry harmonisation across Asia would be valuable for benchmarking outcomes and developing culturally tailored quality-improvement programmes.

This study has several notable strengths. Foremost is its large, multi-ethnic cohort, analysed using prospectively collected registry data over a 5-year period—a duration and scale rarely reported in multi-ethnic Asian populations. Although overall follow-up was 56.4% at 5 years, which is comparable to other long-term registry-based observational cohorts, WOMAC data completeness among responding patients remained high (≥ 98.1% at each interval), supporting the reliability of the patient-reported outcome data among those assessed. Furthermore, capturing standardised PROs at multiple postoperative intervals provided granular insight into the observed recovery pattern and its association with interval-specific predictors. Finally, the real-world, registry-based design enhances generalisability to typical clinical practice in similar healthcare settings.

This study has several limitations. First, the single-centre design may limit generalisability despite the large cohort size and prospective registry structure. Second, we did not capture several important variables; lack of data on radiographic alignment and rehabilitation adherence prevented exploration of their contribution to outcomes, while absence of detailed socioeconomic and psychosocial variables means we could not account for their influence. Third, although analyses revealed no substantial differences between respondents and non-respondents, potential selection bias due to missing data cannot be entirely excluded (Table S6). With 43.6% of the baseline cohort missing at 5 years, patients lost to follow-up may have had systematically poorer outcomes, and the MICE sensitivity analysis conducted under a missing-at-random assumption cannot fully address this concern. Our 5-year results may therefore overestimate improvement in the full population, and mortality status, which was not ascertainable from the registry, may account for a proportion of this attrition. Fourth, exclusive reliance on the WOMAC instrument limits conclusions about other patient-centred domains, such as overall quality of life. Fifth, the dichotomisation of WOMAC change scores at zero, while enabling clear clinical interpretation, may have reduced statistical power by treating small and large improvements equivalently. Sixth, consistent with the paper’s interval-specific framing, our logistic regression models estimate associations at discrete intervals independently and do not attempt to model the shape or evolution of WOMAC change over the full follow-up. A complementary continuous longitudinal analysis (for example, a linear mixed-effects model or a group-based trajectory model, with appropriate attention to the uneven timepoint spacing and attrition mechanisms) would address the shape and subgroup variation of recovery more directly. Seventh, the absence of adjustment for multiple comparisons across four outcomes, four intervals, and multiple covariates means that some observed associations may be attributable to chance. Eighth, the stepwise variable selection approach based on a univariable p < .10 threshold may inflate Type I error; prespecified selection based on clinical rationale would have been more defensible. Ninth, comorbidity was modelled as a binary variable (any versus none), which does not capture the graded effect of comorbidity burden; future studies should consider validated comorbidity indices to characterise this relationship more precisely. observational study, causal inferences cannot be made, and results may be subject to residual confounding.

In this large, multi-ethnic Asian cohort, TKA was associated with significant and sustained WOMAC improvements, with most benefits realised within the first 6 months. Interval-specific predictors of improvement varied by age, comorbidity, and ethnicity, while surgical variables showed limited association. These exploratory findings suggest that patient-level factors may play a more prominent role than technical variables in long-term recovery, supporting the potential value of individualised perioperative strategies and culturally responsive care.

## Supplementary Information

Below is the link to the electronic supplementary material.

Supplementary Material 1

## Data Availability

No datasets were generated or analysed during the current study.
