# Supplementary material for: WOMAC-based recovery after total knee arthroplasty in a multiethnic Asian cohort: a 5-year registry-based study of interval-specific predictors of improvement
Source: Qual Life Res. 2026 May 3;35(6):156. doi: 10.1007/s11136-026-04276-y (PMC13136197; doi:10.1007/s11136-026-04276-y)
Supplement: Supplementary file 1 — Supplementary Material 1 [file 11136_2026_4276_MOESM2_ESM.docx]

**Table S1.** STROBE and RECORD Checklists

|  | **Item No.** | **STROBE items** | **Location in manuscript where items are reported** | **RECORD items** | **Location in manuscript where items are reported** |
| --- | --- | --- | --- | --- | --- |
| **Title and abstract** | | | | | |
|  | 1 | (a) Indicate the study’s design with a commonly used term in the title or the abstract (b) Provide in the abstract an informative and balanced summary of what was done and what was found | Page 1-2 | RECORD 1.1: The type of data used should be specified in the title or abstract. When possible, the name of the databases used should be included.  RECORD 1.2: If applicable, the geographic region and timeframe within which the study took place should be reported in the title or abstract.  RECORD 1.3: If linkage between databases was conducted for the study, this should be clearly stated in the title or abstract. | Page 1-2 |
| **Introduction** | | | | | |
| Background rationale | 2 | Explain the scientific background and rationale for the investigation being reported | Page 3-4 |  |  |
| Objectives | 3 | State specific objectives, including any prespecified hypotheses | Page 3-4 |  |  |
| **Methods** | | | | | |
| Study Design | 4 | Present key elements of study design early in the paper | Page 4 |  |  |
| Setting | 5 | Describe the setting, locations, and relevant dates, including periods of recruitment, exposure, follow-up, and data collection | Page 4 |  |  |
| Participants | 6 | *(a) Cohort study* - Give the eligibility criteria, and the sources and methods of selection of participants. Describe methods of follow-up  *Case-control study* - Give the eligibility criteria, and the sources and methods of case ascertainment and control selection. Give the rationale for the choice of cases and controls  *Cross-sectional study* - Give the eligibility criteria, and the sources and methods of selection of participants  *(b) Cohort study* - For matched studies, give matching criteria and number of exposed and unexposed  *Case-control study* - For matched studies, give matching criteria and the number of controls per case | Page 4 | RECORD 6.1: The methods of study population selection (such as codes or algorithms used to identify subjects) should be listed in detail. If this is not possible, an explanation should be provided.  RECORD 6.2: Any validation studies of the codes or algorithms used to select the population should be referenced. If validation was conducted for this study and not published elsewhere, detailed methods and results should be provided.  RECORD 6.3: If the study involved linkage of databases, consider use of a flow diagram or other graphical display to demonstrate the data linkage process, including the number of individuals with linked data at each stage. | Page 4 |
| Variables | 7 | Clearly define all outcomes, exposures, predictors, potential confounders, and effect modifiers. Give diagnostic criteria, if applicable. | Page 5-6 | RECORD 7.1: A complete list of codes and algorithms used to classify exposures, outcomes, confounders, and effect modifiers should be provided. If these cannot be reported, an explanation should be provided. | Page 5-6 |
| Data sources/ measurement | 8 | For each variable of interest, give sources of data and details of methods of assessment (measurement).  Describe comparability of assessment methods if there is more than one group | Page 5-6 |  |  |
| Bias | 9 | Describe any efforts to address potential sources of bias | Page 11-12 |  |  |
| Study size | 10 | Explain how the study size was arrived at | Page 4 |  |  |
| Quantitative variables | 11 | Explain how quantitative variables were handled in the analyses. If applicable, describe which groupings were chosen, and why | Page 5-6 |  |  |
| Statistical methods | 12 | (a) Describe all statistical methods, including those used to control for confounding  (b) Describe any methods used to examine subgroups and interactions  (c) Explain how missing data were addressed  (d) *Cohort study* - If applicable, explain how loss to follow-up was addressed  *Case-control study* - If applicable, explain how matching of cases and controls was addressed  *Cross-sectional study* - If applicable, describe analytical methods taking account of sampling strategy  (e) Describe any sensitivity analyses | Page 6 |  |  |
| Data access and cleaning methods |  | .. |  | RECORD 12.1: Authors should describe the extent to which the investigators had access to the database population used to create the study population.  RECORD 12.2: Authors should provide information on the data cleaning methods used in the study. | Page 4 |
| Linkage |  | .. |  | RECORD 12.3: State whether the study included person-level, institutional-level, or other data linkage across two or more databases. The methods of linkage and methods of linkage quality evaluation should be provided. | Page 5-6 |
| **Results** | | | | | |
| Participants | 13 | (a) Report the numbers of individuals at each stage of the study (*e.g.*, numbers potentially eligible, examined for eligibility, confirmed eligible, included in the study, completing follow-up, and analysed)  (b) Give reasons for non-participation at each stage.  (c) Consider use of a flow diagram | Page 7 | RECORD 13.1: Describe in detail the selection of the persons included in the study (*i.e.,* study population selection) including filtering based on data quality, data availability and linkage. The selection of included persons can be described in the text and/or by means of the study flow diagram. | Page 7 |
| Descriptive data | 14 | (a) Give characteristics of study participants (*e.g.*, demographic, clinical, social) and information on exposures and potential confounders  (b) Indicate the number of participants with missing data for each variable of interest  (c) *Cohort study* - summarise follow-up time (*e.g.*, average and total amount) | Page 7-8 |  |  |
| Outcome data | 15 | *Cohort study* - Report numbers of outcome events or summary measures over time  *Case-control study* - Report numbers in each exposure category, or summary measures of exposure  *Cross-sectional study* - Report numbers of outcome events or summary measures | Page 7-8 |  |  |
| Main results | 16 | (a) Give unadjusted estimates and, if applicable, confounder-adjusted estimates and their precision (e.g., 95% confidence interval). Make clear which confounders were adjusted for and why they were included  (b) Report category boundaries when continuous variables were categorized  (c) If relevant, consider translating estimates of relative risk into absolute risk for a meaningful time period | Page 7-8 |  |  |
| Other analyses | 17 | Report other analyses done—e.g., analyses of subgroups and interactions, and sensitivity analyses | Page 7-8 |  |  |
| **Discussion** | | | | | |
| Key results | 18 | Summarise key results with reference to study objectives | Page 8-11 |  |  |
| Limitations | 19 | Discuss limitations of the study, taking into account sources of potential bias or imprecision. Discuss both direction and magnitude of any potential bias | Page 8-11 | RECORD 19.1: Discuss the implications of using data that were not created or collected to answer the specific research question(s). Include discussion of misclassification bias, unmeasured confounding, missing data, and changing eligibility over time, as they pertain to the study being reported. | Page 8-11 |
| Interpretation | 20 | Give a cautious overall interpretation of results considering objectives, limitations, multiplicity of analyses, results from similar studies, and other relevant evidence | Page 8-11 |  |  |
| Generalisability | 21 | Discuss the generalisability (external validity) of the study results | Page 8-11 |  |  |
| **Other Information** | | | | | |
| Funding | 22 | Give the source of funding and the role of the funders for the present study and, if applicable, for the original study on which the present article is based | Title Page |  |  |
| Accessibility of protocol, raw data, and programming code |  | .. |  | RECORD 22.1: Authors should provide information on how to access any supplemental information such as the study protocol, raw data, or programming code. | Title Page |

*Reference: Benchimol EI, Smeeth L, Guttmann A, Harron K, Moher D, Petersen I, Sørensen HT, von Elm E, Langan SM, the RECORD Working Committee. The REporting of studies Conducted using Observational Routinely-collected health Data (RECORD) Statement. *PLoS Medicine* 2015; in press.

*Checklist is protected under Creative Commons Attribution ([CC BY](http://creativecommons.org/licenses/by/4.0/)) license.

## Table S2. Categories of sociodemographic and clinical variables

| **Independent variable (s)** | **Categories** |
| --- | --- |
| **Sociodemographic** |  |
| Age, years | Young adult (< 55); Older adult (55-64); Early elder (65-74); Late elder (≥ 75) |
| Gender, M/F | Male; Female |
| Ethnicity | Chinese; Malay; Indian & Others |
| BMI, kg/m^2^ | Underweight (<18.5); Normal (18.5-24.9); Overweight (25-29.9); Obese (≥ 30) |
| **Clinical** |  |
| Presence of ≥1 comorbidity | Yes; No |
| Type of TKA procedure | Bilateral TKA (Both knees); Unilateral and staged TKA (one knee) |
| Surgical time, minutes | ≤ 100; > 100 |
| Tourniquet time, minutes | No tourniquet; ≤ 100; > 100 |
| Type of model implant | Johnson & Johnson (J&J); Smith & Nephew (S&N); Stryker, B Braun & Others (S, B & Others); Zimmer (Z) |
| Post-operative anesthesia | General anesthesia (GA); Non-GA |
| Blood loss, milliliters | <250; ≥250 |
| Thromboembolic prophylaxis | Pharm & Mech; Pharm or Mech; None |
| Post-operative complications | Complication (Prescence of complications); No complication (Absence of complications) |
| Length of stay, days | ≤ 5; > 5 |
| *Note.* This table describes how independent variables in this study (sociodemographic and clinical variables) were categorized into distinct groups to facilitate statistical analysis. Others (Ethnicity): Minority populations that are not part of Singapore’s three main ethnic groups (Chinese, Malay, and Indians); Pharm: Pharmacological thromboembolic prophylaxis that includes use of Low-Molecular-Weight-Heparins (LMWHs), heparins, and aspirin to prevent post-operative Venous thromboembolism (VTE); Mech: Mechanical thromboembolic prophylaxis that includes compression devices such as thromboembolic deterrent stockings (TEDs), calf pumps and foot pumps to prevent post-operative VTE. | |

##

## Table S3. Univariate analysis of WOMAC total and subscale scores

| **Variables** | **T_0_-T_1_ (N = 4,694)** | | **T_1_-T_2_ (N = 4,431)** | | **T_2_-T_3_ (N = 3,919)** | | **T_3_-T_4_ (N = 2,749)** | |
| --- | --- | --- | --- | --- | --- | --- | --- | --- |
|  | **N^a^ (%)** | ***p*-value^b^** | **N^a^ (%)** | ***p*-value^b^** | **N^a^ (%)** | ***p*-value^b^** | **N^a^ (%)** | ***p*-value^b^** |
| **Total, N^c^** | 4503 (95.9) |  | 2454 (55.4) |  | 2147 (54.8) |  | 1366 (49.7) |  |
| **Age, years** |  |  |  |  |  |  |  |  |
| Young adult (<55) | 289 (96.0) | 0.299 | 168 (60.4) | 0.246 | 145 (54.5) | 0.687 | 107 (49.8) | 0.037* |
| Older adult (55-64) | 1422 (96.3) |  | 779 (56.1) |  | 668 (53.4) |  | 470 (49.9) |  |
| Early elder (65-74) | 1997 (96.1) |  | 1085 (54.8) |  | 976 (55.6) |  | 607 (51.7) |  |
| Late elder (≥75) | 795 (94.8) |  | 422 (53.9) |  | 358 (55.2) |  | 182 (43.4) |  |
| **Gender** |  |  |  |  |  |  |  |  |
| Female | 3150 (96.5) | 0.003** | 1718 (55.8) | 0.402 | 1495 (54.6) | 0.670 | 983 (50.1) | 0.470 |
| Male | 1353 (94.6) |  | 736 (54.4) |  | 652 (55.3) |  | 383 (48.6) |  |
| **Ethnicity** |  |  |  |  |  |  |  |  |
| Chinese | 3228 (96.0) | 0.742 | 1766 (55.1) | 0.849 | 1538 (54.3) | 0.570 | 1003 (49.8) | 0.968 |
| Malay | 676 (95.9) |  | 368 (55.8) |  | 327 (55.4) |  | 190 (49.1) |  |
| Indian and others | 599 (95.4) |  | 320 (56.3) |  | 282 (56.7) |  | 173 (49.9) |  |
| **BMI, kg/m^2^** |  |  |  |  |  |  |  |  |
| Underweight (<18.5) | 35 (94.6) | 0.735 | 16 (47.1) | 0.804 | 13 (44.8) | 0.455 | 9 (40.9) | 0.649 |
| Normal (18.5-24.9) | 1275 (95.7) |  | 696 (55.3) |  | 597 (53.4) |  | 382 (48.3) |  |
| Overweight (25-29.9) | 1876 (95.8) |  | 1031 (55.6) |  | 902 (55.2) |  | 573 (50.4) |  |
| Obese (≥ 30) | 1317 (96.4) |  | 711 (55.4) |  | 635 (55.8) |  | 402 (50.3) |  |
| **Presence of ≥1 comorbidity** |  |  |  |  |  |  |  |  |
| Yes | 3501 (96.3) | 0.014* | 1870 (54.3) | 0.008** | 1659 (55.0) | 0.669 | 1032 (49.1) | 0.261 |
| No | 1002 (94.6) |  | 584 (59.1) |  | 488 (54.2) |  | 334 (51.6) |  |
| **Type of TKA procedure** |  |  |  |  |  |  |  |  |
| Bilateral TKA | 151 (95.0) | 0.532 | 85 (58.6) | 0.427 | 78 (61.4) | 0.126 | 36 (45.6) | 0.455 |
| Unilateral and staged TKA | 4351 (96.0) |  | 2369 (55.3) |  | 2068 (54.6) |  | 1330 (49.8) |  |
| **Surgical time, mins** |  |  |  |  |  |  |  |  |
| ≤100 | 1789 (96.4) | 0.151 | 1006 (57.4) | 0.032* | 839 (55.3) | 0.611 | 503 (46.7) | 0.011* |
| >100 | 2712 (95.6) |  | 1448 (54.1) |  | 1306 (54.4) |  | 863 (51.6) |  |
| **Tourniquet time, mins** |  |  |  |  |  |  |  |  |
| No tourniquet | 44 (95.7) | 0.980 | 22 (50.0) | 0.176 | 22 (55.0) | 0.771 | 20 (66.7) | 0.003** |
| ≤100 | 2935 (95.9) |  | 1631 (56.4) |  | 1379 (54.5) |  | 831 (47.4) |  |
| >100 | 1462 (96.0) |  | 772 (53.7) |  | 730 (55.7) |  | 501 (53.7) |  |
| **Type of implant model** |  |  |  |  |  |  |  |  |
| Model J&J | 877 (95.3) | 0.257 | 471 (54.0) | 0.635 | 452 (57.1) | 0.214 | 294 (51.3) | <.001*** |
| Model S&N | 1090 (96.1) |  | 612 (56.6) |  | 499 (56.3) |  | 298 (63.7) |  |
| Models S, B & Others | 770 (95.1) |  | 416 (54.2) |  | 385 (53.9) |  | 295 (48.7) |  |
| Model Z | 1764 (96.5) |  | 955 (55.8) |  | 810 (53.1) |  | 479 (43.5) |  |
| **Type of anesthesia** |  |  |  |  |  |  |  |  |
| GA | 3603 (96.0) | 0.487 | 1963 (55.5) | 0.723 | 1747 (54.7) | 0.942 | 1167 (49.1) | 0.115 |
| Non-GA | 897 (95.5) |  | 490 (54.9) |  | 398 (54.9) |  | 199 (53.5) |  |
| **Blood loss, ml (s)** |  |  |  |  |  |  |  |  |
| <250 | 3773 (95.8) | 0.764 | 2064 (55.6) | 0.517 | 1889 (54.5) | 0.381 | 1190 (49.4) | 0.361 |
| ≥250 | 472 (95.6) |  | 271 (57.2) |  | 244 (56.7) |  | 176 (52.1) |  |
| **TP** |  |  |  |  |  |  |  |  |
| Pharm & Mech | 2633 (95.5) | 0.041* | 1461 (55.4) | 0.486 | 1258 (55.8) | 0.306 | 763 (57.2) | <.001*** |
| Pharm or Mech | 1806 (96.7) |  | 953 (55.1) |  | 860 (53.4) |  | 586 (42.9) |  |
| None | 58 (92.1) |  | 36 (63.2) |  | 28 (52.8) |  | 17 (34.7) |  |
| **Post-op complication** |  |  |  |  |  |  |  |  |
| No complication | 3554 (95.9) | 0.525 | 1980 (56.5) | 0.140 | 1784 (54.6) | 0.612 | 1155 (50.6) | 0.045* |
| Complication | 655 (95.3) |  | 345 (53.3) |  | 339 (55.8) |  | 200 (45.4) |  |
| **Length of stay, days** |  |  |  |  |  |  |  |  |
| **≤** 5 | 3342 (95.9) | 0.929 | 1798 (54.6) | 0.066 | 1569 (54.7) | 0.941 | 986 (52.3) | <.001*** |
| **>** 5 | 1147 (96.0) |  | 650 (55.4) |  | 570 (54.9) |  | 375 (43.8) |  |
| **Stiffness, N^c^** | 2956 (63.0) |  | 1219 (27.5) |  | 857 (21.9) |  | 461 (16.8) |  |
| **Age, years** |  |  |  |  |  |  |  |  |
| Young adult (<55) | 203 (67.4) | 0.359 | 81 (29.1) | 0.796 | 62 (23.3) | 0.439 | 49 (22.8) | 0.107 |
| Older adult (55-64) | 933 (63.2) |  | 375 (27.0) |  | 264 (21.1) |  | 154 (16.4) |  |
| Early elder (65-74) | 1302 (62.7) |  | 554 (28.0) |  | 400 (22.8) |  | 189 (16.1) |  |
| Late elder (≥75) | 518 (61.7) |  | 209 (26.7) |  | 131 (20.2) |  | 69 (16.5) |  |
| **Gender** |  |  |  |  |  |  |  |  |
| Female | 2094 (64.2) | 0.011* | 878 (28.5) | 0.024* | 608 (22.2) | 0.457 | 339 (17.3) | 0.252 |
| Male | 862 (60.3) |  | 341 (25.2) |  | 249 (21.1) |  | 122 (15.5) |  |
| **Ethnicity** |  |  |  |  |  |  |  |  |
| Chinese | 2124 (63.2) | 0.750 | 891 (27.8) | 0.467 | 641 (22.6) | 0.128 | 326 (16.2) | 0.021* |
| Malay | 435 (61.7) |  | 184 (27.9) |  | 112 (19.0) |  | 59 (15.3) |  |
| Indian and others | 397 (63.2) |  | 144 (25.4) |  | 104 (20.9) |  | 76 (21.9) |  |
| **BMI, kg/m^2^** |  |  |  |  |  |  |  |  |
| Underweight (<18.5) | 24 (64.9) | 0.232 | 4 (11.8) | 0.217 | 5 (17.2) | 0.818 | 3 (13.6) | 0.831 |
| Normal (18.5-24.9) | 848 (63.7) |  | 345 (27.4) |  | 237 (21.2) |  | 130 (16.4) |  |
| Overweight (25-29.9) | 1201 (61.3) |  | 519 (28.0) |  | 359 (22.0) |  | 199 (17.5) |  |
| Obese (≥ 30) | 883 (64.6) |  | 351 (27.4) |  | 256 (22.5) |  | 129 (16.2) |  |
| **Presence of ≥1 comorbidity** |  |  |  |  |  |  |  |  |
| Yes | 2250 (61.9) | 0.005** | 940 (27.3) | 0.561 | 675 (22.4) | 0.167 | 342 (16.3) | 0.206 |
| No | 706 (66.7) |  | 279 (28.2) |  | 182 (20.2) |  | 119 (18.4) |  |
| **Type of TKA procedure** |  |  |  |  |  |  |  |  |
| Bilateral TKA | 106 (66.7) | 0.326 | 32 (22.1) | 0.135 | 31 (24.4) | 0.482 | 20 (25.3) | 0.039* |
| Unilateral and staged TKA | 2849 (62.8) |  | 1187 (27.7) |  | 826 (21.8) |  | 441 (16.5) |  |
| **Surgical time, mins** |  |  |  |  |  |  |  |  |
| ≤100 | 1161 (62.6) | 0.653 | 500 (28.5) | 0.218 | 337 (22.2) | 0.699 | 186 (17.3) | 0.578 |
| >100 | 1794 (63.2) |  | 718 (26.8) |  | 520 (21.7) |  | 275 (16.5) |  |
| **Tourniquet time, mins** |  |  |  |  |  |  |  |  |
| No tourniquet | 27 (58.7) | 0.236 | 5 (11.4) | 0.049* | 6 (15.0) | 0.375 | 2 (6.7) | 0.132 |
| ≤100 | 1951 (63.7) |  | 795 (27.5) |  | 566 (22.4) |  | 308 (17.6) |  |
| >100 | 934 (61.3) |  | 405 (28.2) |  | 276 (21.1) |  | 146 (15.5) |  |
| **Type of implant model** |  |  |  |  |  |  |  |  |
| Model J&J | 577 (62.7) | <0.001*** | 223 (25.6) | 0.410 | 176 (22.3) | 0.001** | 93 (16.2) | 0.413 |
| Model S&N | 740 (65.3) |  | 307 (28.4) |  | 225 (25.4) |  | 78 (16.7) |  |
| Models S, B & Others | 447 (55.2) |  | 204 (26.6) |  | 169 (23.7) |  | 115 (19.0) |  |
| Model Z | 1190 (65.1) |  | 485 (28.4) |  | 287 (18.8) |  | 175 (15.9) |  |
| **Type of anesthesia** |  |  |  |  |  |  |  |  |
| GA | 2348 (62.6) | 0.294 | 967 (27.4) | 0.702 | 695 (21.8) | 0.740 | 399 (16.8) | 0.954 |
| Non-GA | 605 (64.4) |  | 250 (28.0) |  | 162 (22.3) |  | 62 (16.7) |  |
| **Blood loss, ml (s)** |  |  |  |  |  |  |  |  |
| <250 | 2437 (61.9) | 0.473 | 1045 (28.2) | 0.060 | 763 (22.0) | 0.532 | 419 (17.4) | 0.015* |
| ≥250 | 314 (63.6) |  | 114 (24.1) |  | 89 (20.7) |  | 41 (12.1) |  |
| **TP** |  |  |  |  |  |  |  |  |
| Pharm & Mech | 1773 (64.3) | 0.024* | 720 (27.3) | 0.751 | 511 (22.7) | 0.219 | 211 (15.8) | 0.427 |
| Pharm or Mech | 1134 (60.7) |  | 479 (27.7) |  | 338 (21.0) |  | 241 (17.6) |  |
| None | 44 (69.8) |  | 18 (31.6) |  | 8 (15.1) |  | 9 (18.4) |  |
| **Post-op complication** |  |  |  |  |  |  |  |  |
| No complication | 2312 (62.4) | 0.182 | 976 (27.8) | 0.577 | 695 (21.3) | 0.159 | 395 (17.3) | 0.232 |
| Complication | 410 (59.7) |  | 187 (28.9) |  | 145 (23.9) |  | 66 (15.0) |  |
| **Length of stay, days** |  |  |  |  |  |  |  |  |
| **≤** 5 | 2191 (62.9) | 0.938 | 901 (27.4) | 0.600 | 616 (21.5) | 0.376 | 308 (16.3) | 0.354 |
| **>** 5 | 753 (63.0) |  | 317 (28.2) |  | 237 (22.8) |  | 152 (17.8) |  |
| **Pain, N^c^** | 4490 (95.7) |  | 1167 (26.3) |  | 743 (19.0) |  | 418 (15.2) |  |
| **Age, years** |  |  |  |  |  |  |  |  |
| Young adult (<55) | 290 (96.4) | 0.419 | 79 (28.4) | 0.518 | 44 (16.5) | 0.150 | 35 (16.3) | 0.380 |
| Older adult (55-64) | 1421 (96.2) |  | 376 (27.1) |  | 242 (19.4) |  | 141 (15.0) |  |
| Early elder (65-74) | 1983 (95.5) |  | 501 (25.3) |  | 351 (20.0) |  | 189 (16.1) |  |
| Late elder (≥75) | 796 (94.9) |  | 211 (27.0) |  | 106 (16.3) |  | 53 (12.6) |  |
| **Gender** |  |  |  |  |  |  |  |  |
| Female | 3133 (96.0) | 0.091 | 804 (26.1) | 0.608 | 540 (19.7) | 0.068 | 294 (15.0) | 0.623 |
| Male | 1357 (94.9) |  | 363 (26.9) |  | 203 (17.2) |  | 124 (15.7) |  |
| **Ethnicity** |  |  |  |  |  |  |  |  |
| Chinese | 3210 (95.5) | 0.197 | 800 (25.0) | 0.001** | 511 (18.0) | 0.004** | 287 (14.2) | 0.008** |
| Malay | 683 (96.9) |  | 185 (28.0) |  | 111 (18.8) |  | 59 (15.3) |  |
| Indian and others | 597 (95.1) |  | 182 (32.0) |  | 121 (24.4) |  | 72 (20.8) |  |
| **BMI, kg/m^2^** |  |  |  |  |  |  |  |  |
| Underweight (<18.5) | 35 (94.6) | 0.214 | 7 (20.6) | 0.101 | 5 (17.2) | 0.739 | 5 (22.7) | 0.706 |
| Normal (18.5-24.9) | 1269 (95.3) |  | 303 (24.1) |  | 200 (17.9) |  | 120 (15.2) |  |
| Overweight (25-29.9) | 1866 (95.3) |  | 496 (26.7) |  | 318 (19.5) |  | 167 (14.7) |  |
| Obese (≥ 30) | 1320 (96.6) |  | 361 (28.1) |  | 220 (19.3) |  | 126 (15.8) |  |
| **Presence of ≥1 comorbidity** |  |  |  |  |  |  |  |  |
| Yes | 3487 (95.9) | 0.088 | 914 (26.6) | 0.555 | 580 (19.2) | 0.449 | 96 (14.8) | 0.766 |
| No | 1003 (94.7) |  | 253 (25.6) |  | 163 (18.1) |  | 322 (15.3) |  |
| **Type of TKA procedure** |  |  |  |  |  |  |  |  |
| Bilateral TKA | 153 (96.2) | 0.718 | 39 (26.9) | 0.878 | 22 (17.3) | 0.632 | 10 (12.7) | 0.521 |
| Unilateral and staged TKA | 4336 (95.6) |  | 1128 (26.3) |  | 721 (19.0) |  | 408 (15.3) |  |
| **Surgical time, mins** |  |  |  |  |  |  |  |  |
| ≤100 | 1777 (95.8) | 0.698 | 465 (26.5) | 0.829 | 301 (19.8) | 0.260 | 151 (14.0) | 0.163 |
| >100 | 2711 (95.6) |  | 702 (26.2) |  | 441 (18.4) |  | 267 (16.0) |  |
| **Tourniquet time, mins** |  |  |  |  |  |  |  |  |
| No tourniquet | 46 (100.0) | 0.170 | 9 (20.5) | 0.253 | 6 (15.0) | 0.814 | 3 (10.0) | 0.727 |
| ≤100 | 2919 (95.4) |  | 740 (25.6) |  | 481 (19.0) |  | 268 (15.3) |  |
| >100 | 1464 (96.1) |  | 397 (27.6) |  | 249 (19.0) |  | 143 (15.2) |  |
| **Type of implant model** |  |  |  |  |  |  |  |  |
| Model J&J | 884 (96.1) | 0.186 | 239 (27.5) | 0.638 | 137 (17.3) | 0.053 | 84 (14.7) | 0.832 |
| Model S&N | 1094 (96.5) |  | 288 (26.6) |  | 186 (21.0) |  | 72 (15.4) |  |
| Models S, B & Others | 766 (94.6) |  | 207 (27.0) |  | 151 (21.2) |  | 87 (14.4) |  |
| Model Z | 1744 (95.4) |  | 433 (25.3) |  | 269 (17.6) |  | 175 (15.9) |  |
| **Type of anesthesia** |  |  |  |  |  |  |  |  |
| GA | 3583 (95.5) | 0.297 | 919 (26.0) | 0.314 | 604 (18.9) | 0.880 | 350 (14.7) | 0.076 |
| Non-GA | 904 (96.3) |  | 247 (27.7) |  | 139 (19.2) |  | 68 (18.3) |  |
| **Blood loss, ml (s)** |  |  |  |  |  |  |  |  |
| <250 | 3760 (95.5) | 0.966 | 960 (25.9) | 0.087 | 656 (18.9) | 0.962 | 375 (15.6) | 0.172 |
| ≥250 | 472 (95.6) |  | 140 (29.5) |  | 81 (18.8) |  | 43 (12.7) |  |
| **TP** |  |  |  |  |  |  |  |  |
| Pharm & Mech | 2637 (95.7) | 0.731 | 725 (27.5) | 0.012* | 416 (18.5) | 0.308 | 212 (15.9) | 0.560 |
| Pharm or Mech | 1788 (95.7) |  | 434 (25.1) |  | 320 (19.9) |  | 200 (14.6) |  |
| None | 59 (93.7) |  | 7 (12.3) |  | 7 (13.2) |  | 6 (12.2) |  |
| **Post-op complication** |  |  |  |  |  |  |  |  |
| No complication | 3534 (95.3) | 0.496 | 919 (26.2) | 0.970 | 608 (18.6) | 0.134 | 346 (15.2) | 0.826 |
| Complication | 659 (95.9) |  | 170 (26.3) |  | 129 (21.2) |  | 65 (14.7) |  |
| **Length of stay, days** |  |  |  |  |  |  |  |  |
| **≤** 5 | 3337 (95.8) | 0.494 | 853 (25.9) | 0.306 | 539 (18.8) | 0.603 | 269 (14.3) | 0.041* |
| **>** 5 | 1139 (95.3) |  | 309 (27.5) |  | 203 (19.5) |  | 148 (17.3) |  |
| **Physical function, N^c^** | 4411 (94.0) |  | 2368 (53.4) |  | 2097 (53.5) |  | 1391 (50.6) |  |
| **Age, years** |  |  |  |  |  |  |  |  |
| Young adult (<55) | 281 (93.4) | 0.063 | 159 (57.2) | 0.572 | 142 (53.4) | 0.844 | 108 (50.2) | 0.066 |
| Older adult (55-64) | 1403 (95.0) |  | 747 (53.8) |  | 656 (52.5) |  | 472 (50.2) |  |
| Early elder (65-74) | 1953 (94.0) |  | 1050 (53.0) |  | 948 (54.1) |  | 621 (52.9) |  |
| Late elder (≥75) | 774 (92.3) |  | 412 (52.6) |  | 351 (54.1) |  | 190 (45.4) |  |
| **Gender** |  |  |  |  |  |  |  |  |
| Female | 3084 (94.5) | 0.025* | 1649 (53.6) | 0.817 | 1469 (53.6) | 0.841 | 994 (50.7) | 0.884 |
| Male | 1327 (92.8) |  | 719 (53.2) |  | 628 (53.3) |  | 397 (50.4) |  |
| **Ethnicity** |  |  |  |  |  |  |  |  |
| Chinese | 3156 (93.9) | 0.587 | 1712 (53.5) | 0.815 | 1502 (53.0) | 0.527 | 1035 (51.4) | 0.391 |
| Malay | 668 (94.8) |  | 347 (52.6) |  | 318 (53.9) |  | 190 (49.1) |  |
| Indian and others | 587 (93.5) |  | 309 (54.4) |  | 277 (55.7) |  | 166 (47.8) |  |
| **BMI, kg/m^2^** |  |  |  |  |  |  |  |  |
| Underweight (<18.5) | 35 (94.6) | 0.321 | 17 (50.0) | 0.924 | 13 (44.8) | 0.649 | 9 (40.9) | 0.604 |
| Normal (18.5-24.9) | 1249 (93.8) |  | 669 (53.1) |  | 587 (52.5) |  | 389 (49.2) |  |
| Overweight (25-29.9) | 1830 (93.4) |  | 1001 (54.0) |  | 885 (54.2) |  | 582 (51.2) |  |
| Obese (≥ 30) | 1297 (95.0) |  | 681 (53.1) |  | 612 (53.8) |  | 411 (51.4) |  |
| **Presence of ≥1 comorbidity** |  |  |  |  |  |  |  |  |
| Yes | 3437 (94.6) | 0.002** | 1803 (52.4) | 0.007** | 1620 (53.7) | 0.697 | 332 (51.3) | 0.678 |
| No | 974 (92.0) |  | 565 (57.2) |  | 477 (52.9) |  | 1059 (50.4) |  |
| **Type of TKA procedure** |  |  |  |  |  |  |  |  |
| Bilateral TKA | 146 (91.8) | 0.248 | 88 (60.7) | 0.076 | 74 (58.3) | 0.273 | 34 (43.0) | 0.171 |
| Unilateral and staged TKA | 4264 (94.0) |  | 2280 (53.2) |  | 2022 (53.3) |  | 1357 (50.8) |  |
| **Surgical time, mins** |  |  |  |  |  |  |  |  |
| ≤100 | 1740 (93.8) | 0.696 | 965 (55.1) | 0.087 | 812 (53.5) | 0.995 | 513 (47.6) | 0.012* |
| >100 | 2669 (94.1) |  | 1403 (52.4) |  | 1283 (53.5) |  | 878 (52.5) |  |
| **Tourniquet time, mins** |  |  |  |  |  |  |  |  |
| No tourniquet | 43 (93.5) | 0.986 | 21 (47.7) | 0.114 | 21 (52.5) | 0.443 | 20 (66.7) | 0.003** |
| ≤100 | 2876 (94.0) |  | 1581 (54.7) |  | 1338 (52.8) |  | 848 (48.3) |  |
| >100 | 1432 (94.0) |  | 742 (51.6) |  | 721 (55.0) |  | 510 (54.1) |  |
| **Type of implant model** |  |  |  |  |  |  |  |  |
| Model J&J | 852 (92.6) | 0.018* | 448 (51.5) | 0.602 | 451 (57.0) | 0.082 | 301 (52.5) | <0.001 *** |
| Model S&N | 1072 (94.5) |  | 586 (54.2) |  | 473 (53.3) |  | 299 (63.9) |  |
| Models S, B & Others | 749 (92.5) |  | 408 (53.2) |  | 360 (50.4) |  | 303 (50.0) |  |
| Model Z | 1736 (95.0) |  | 925 (54.1) |  | 812 (53.3) |  | 488 (44.3) |  |
| **Type of anesthesia** |  |  |  |  |  |  |  |  |
| GA | 3520 (93.8) | 0.387 | 1899 (53.7) | 0.521 | 1707 (53.5) | 0.991 | 1192 (50.2) | 0.23 |
| Non-GA | 888 (94.6) |  | 469 (52.5) |  | 388 (53.5) |  | 199 (53.5) |  |
| **Blood loss, ml (s)** |  |  |  |  |  |  |  |  |
| <250 | 3695 (93.9) | 0.643 | 1987 (53.5) | 0.250 | 1850 (53.4) | 0.687 | 1208 (50.2) | 0.171 |
| ≥250 | 461 (93.3) |  | 267 (56.3) |  | 234 (54.4) |  | 183 (54.1) |  |
| **TP** |  |  |  |  |  |  |  |  |
| Pharm & Mech | 2575 (93.4) | 0.044* | 1404 (53.2) | 0.921 | 1209 (53.7) | 0.800 | 775 (58.1) | <0.001*** |
| Pharm or Mech | 1774 (95.0) |  | 930 (53.8) |  | 861 (53.5) |  | 597 (43.7) |  |
| None | 57 (90.5) |  | 31 (54.4) |  | 26 (49.1) |  | 19 (38.8) |  |
| **Post-op complication** |  |  |  |  |  |  |  |  |
| No complication | 3481 (93.9) | 0.303 | 1906 (54.4) | 0.257 | 1741 (53.3) | 0.561 | 1176 (51.5) | 0.066 |
| Complication | 638 (92.9) |  | 336 (51.9) |  | 332 (54.6) |  | 206 (46.7) |  |
| **Length of stay, days** |  |  |  |  |  |  |  |  |
| **≤** 5 | 3277 (94.1) | 0.753 | 1746 (53.0) | 0.319 | 1536 (53.6) | 0.846 | 985 (52.2) | 0.009** |
| **>** 5 | 1121 (94.1) |  | 616 (54.8) |  | 553 (53.2) |  | 401 (46.9) |  |
| T_0_: Pre-op (baseline). T_1_: 6 months follow-up. T_2_: 1 year follow-up. T_3_: 2-year follow-up. T_4_: 5-year follow-up; N^a^ (%): Frequency (percentage) of patients who showed improvement in WOMAC score; *p*-value^b^: P-value for Pearson’s chi-square test of independence; N^c^: Total number of patients who showed improvement in the cohort; Others (Ethnicity): Minority populations that are not part of Singapore’s three main ethnic groups (Chinese, Malay, and Indians); TP: Thromboembolic prophylaxis; Pharm: Pharmacological thromboembolic prophylaxis that includes use of Low-Molecular-Weight-Heparins (LMWHs), heparins, and aspirin to prevent post-operative Venous thromboembolism (VTE); Mech: Mechanical thromboembolic prophylaxis that includes compression devices such as thromboembolic deterrent stockings (TEDs), calf pumps and foot pumps to prevent post-operative VTE.  **p*-value < 0.05; ***p*-value < 0.01; ****p*-value < .001. | | | | | | | | |

**Table S4.** Multivariable mixed-effects logistic analysis of WOMAC total and subscale scores with a random intercept for surgeon, and adjusted for year of surgery and baseline severity as fixed effects

| **Variables** | **T_0_-T_1_** |  | **T_1_-T_2_** |  | **T_2_-T_3_** |  | **T_3_-T_4_** |
| --- | --- | --- | --- | --- | --- | --- | --- |
|  | **OR^c^ (95% CI)** |  | **OR^c^ (95% CI)** |  | **OR^c^ (95% CI)** |  | **OR^c^ (95% CI)** |
| **Total WOMAC score** |  |  |  |  |  |  |  |
| ***Variables of interests*** |  |  |  |  |  |  |  |
| **Age, years (Ref: Young adult (<55))** |  |  |  |  |  |  |  |
| Older adult (55-64) |  |  |  |  |  |  | 0.95 (0.69, 1.31) |
| Early elder (65-74) |  |  |  |  |  |  | 0.84 (0.62, 1.16) |
| Late elder (≥75) |  |  |  |  |  |  | 0.55 (0.39, 0.79)** |
| **Gender (Reference: Female)** |  |  |  |  |  |  |  |
| Male | 0.90 (0.66, 1.23) |  |  |  |  |  |  |
| **Presence of ≥ 1 comorbidity (Ref: No)** |  |  |  |  |  |  |  |
| Yes | 1.24 (0.89, 1.73) |  | 0.83 (0.71, 0.96)* |  |  |  |  |
| **Surgical time, mins (Ref: ≤100)** |  |  |  |  |  |  |  |
| >100 |  |  | 0.92 (0.80, 1.05) |  |  |  | 0.99 (0.83, 1.18) |
| **Tourniquet time, mins (Ref: No or ≤100)** |  |  |  |  |  |  |  |
| >100 |  |  | 0.92 (0.80, 1.06) |  |  |  | 1.09 (0.91, 1.30) |
| **Type of implant model (Ref:** **Model J&J)** |  |  |  |  |  |  |  |
| Model S&N |  |  |  |  |  |  | 1.11 (0.85, 1.45) |
| Model Z |  |  |  |  |  |  | 0.79 (0.63, 1.00) |
| Models S, B & Others |  |  |  |  |  |  | 0.95 (0.73, 1.25) |
| **TP (Ref:** **Pharm & Mech)** |  |  |  |  |  |  |  |
| Pharm or Mech or None | 1.37 (0.95, 1.98) |  |  |  |  |  | 1.18 (0.95, 1.47) |
| **Postoperative complications (Ref: No)** |  |  |  |  |  |  |  |
| Yes |  |  |  |  |  |  | 0.85 (0.68, 1.05) |
| **Length of stay, days (Ref: ≤ 5)** |  |  |  |  |  |  |  |
| > 5 |  |  | 1.13 (0.98, 1.30) |  |  |  | 0.97 (0.81, 1.16) |
| ***Adjustment variables*** |  |  |  |  |  |  |  |
| **Baseline WOMAC total** | 1.10 (1.09, 1.12) |  | 1.00 (1.00, 1.01) |  | 1.00 (1.00, 1.00) |  | 1.00 (1.00, 1.01) |
| **Year of surgery** | 0.99 (0.94, 1.04) |  | 1.00 (0.98, 1.02) |  | 1.03 (1.01, 1.05) |  | 1.25 (1.21, 1.29) |
| **Surgeon random effect variance** | <0.01 |  | 0.004 |  | 0.005 |  | <0.01 |
| **Model fit (Hat^2^ p-value)** | <0.001 |  | 0.90 |  | - |  | 0.55 |
| **Stiffness subscale score** |  |  |  |  |  |  |  |
| ***Variables of interests*** |  |  |  |  |  |  |  |
| **Gender (Reference: Female)** |  |  |  |  |  |  |  |
| Male | 1.18 (0.97, 1.44) |  | 0.86 (0.74, 1.00)* |  |  |  |  |
| **Ethnicity (Ref: Chinese)** |  |  |  |  |  |  |  |
| Malay |  |  |  |  |  |  | 0.92 (0.68, 1.24) |
| Indian or others |  |  |  |  |  |  | 1.45 (1.10, 1.93)* |
| **Presence of ≥ 1 comorbidity (Ref: No)** |  |  |  |  |  |  |  |
| Yes | 0.90 (0.72, 1.12) |  |  |  |  |  |  |
| **Type of TKA procedure (Ref: Bilateral TKA)** |  |  |  |  |  |  |  |
| Unilateral and staged TKA |  |  |  |  |  |  | 0.53 (0.31, 0.91)* |
| **Blood loss, ml (s) (Ref: <250)** |  |  |  |  |  |  |  |
| ≥250 |  |  | 0.81 (0.65, 1.01) |  |  |  | 0.62 (0.44, 0.89)** |
| **Type of implant model (Ref:** **Model J&J)** |  |  |  |  |  |  |  |
| Model S&N | 0.94 (0.69, 1.26) |  |  |  | 1.17 (0.93, 1.48) |  |  |
| Model Z | 1.05 (0.78, 1.41) |  |  |  | 0.82 (0.66, 1.01) |  |  |
| Models S, B & Others | 0.72 (0.50, 1.03) |  |  |  | 1.14 (0.89, 1.45) |  |  |
| **TP (Ref:** **Pharm & Mech)** |  |  |  |  |  |  |  |
| Pharm or Mech or None | 0.95 (0.74, 1.23) |  |  |  |  |  |  |
| ***Adjustment variables*** |  |  |  |  |  |  |  |
| **Baseline stiffness score** | 16.20 (13.90, 18.87) |  | 1.15 (1.07, 1.23) |  | 1.17 (1.08, 1.26) |  | 1.24 (1.12, 1.37) |
| **Year of surgery** | 0.93 (0.90, 0.96) |  | 1.00 (0.98, 1.02) |  | 1.01 (0.98, 1.03) |  | 0.99 (0.96, 1.02) |
| **Surgeon random effect variance** | 0.01 |  | 0.009 |  | <0.01 |  | <0.01 |
| **Model fit (Hat^2^ p-value)** | <0.001 |  | 0.40 |  | 0.68 |  | 0.36 |
| **Pain subscale score** |  |  |  |  |  |  |  |
| ***Variables of interests*** |  |  |  |  |  |  |  |
| **Gender (Reference: Female)** |  |  |  |  |  |  |  |
| Male | 0.97 (0.70, 1.35) |  |  |  | 0.88 (0.73, 1.05) |  |  |
| **Ethnicity (Ref: Chinese)** |  |  |  |  |  |  |  |
| Malay |  |  | 1.06 (0.86, 1.30) |  | 1.35 (0.70, 2.59) |  | 0.98 (0.72, 1.34) |
| Indian or others |  |  | 1.35 (1.10, 1.66)** |  | 2.87 (1.62, 5.08)*** |  | 1.45 (1.08, 1.94)* |
| **BMI, kg/m^2^ (Ref: Obese (≥ 30))** |  |  |  |  |  |  |  |
| Underweight (<18.5) |  |  | 0.67 (0.27, 1.64) |  |  |  |  |
| Normal (18.5- 24.9) |  |  | 0.90 (0.76, 1.07) |  |  |  |  |
| Overweight (25-29.9) |  |  | 1.01 (0.85, 1.20) |  |  |  |  |
| **Presence of ≥ 1 comorbidity (Ref: No)** |  |  |  |  |  |  |  |
| Yes | 1.16 (0.81, 1.65) |  |  |  |  |  |  |
| **Type of anesthesia (Ref: GA)** |  |  |  |  |  |  |  |
| Non-GA |  |  |  |  |  |  | 1.26 (0.94, 1.68) |
| **Blood loss, ml (s) (Ref: <250)** |  |  |  |  |  |  |  |
| ≥250 |  |  | 1.21 (0.98, 1.49) |  |  |  |  |
| **Type of implant model (Ref:** **Model J&J)** |  |  |  |  |  |  |  |
| Model S&N |  |  |  |  | 1.30 (1.01, 1.68)* |  |  |
| Model Z |  |  |  |  | 0.98 (0.78, 1.23) |  |  |
| Models S, B & Others |  |  |  |  | 1.21 (0.93, 1.57) |  |  |
| **TP (Ref:** **Pharm & Mech)** |  |  |  |  |  |  |  |
| Pharm or Mech or None |  |  | 0.88 (0.74, 1.05) |  |  |  |  |
| **Length of stay, days (Ref: ≤ 5)** |  |  |  |  |  |  |  |
| > 5 |  |  |  |  |  |  | 1.31 (1.04, 1.65)* |
| **Ethnicity *Baseline pain score (Ref: Chinese)** |  |  |  |  |  |  |  |
| Malay |  |  |  |  | 0.82 (0.58, 1.17) |  |  |
| Indian or others |  |  |  |  | 0.64 (0.46, 0.89)** |  |  |
| ***Adjustment variables*** |  |  |  |  |  |  |  |
| **Baseline pain score** | 19.76 (13.88, 28.14) |  | 1.23 (1.11, 1.37) |  | 1.36 (1.17, 1.58) |  | 1.39 (1.19, 1.63) |
| **Year of surgery** | 1.01 (0.97, 1.05) |  | 1.01 (0.98, 1.03) |  | 0.99 (0.97, 1.01) |  | 1.03 (0.99, 1.06) |
| **Surgeon random effect variance** | 0.031 |  | 0.001 |  | <0.01 |  | <0.01 |
| **Model fit (Hat^2^ p-value)** | <0.001 |  | 0.07 |  | 0.79 |  | 0.43 |
| **Physical function subscale score** |  |  |  |  |  |  |  |
| ***Variables of interests*** |  |  |  |  |  |  |  |
| **Age, years (Ref: Young adult (<55))** |  |  |  |  |  |  |  |
| Older adult (55-64) | 1.56 (0.91, 2.67) |  |  |  |  |  | 0.95 (0.69, 1.31) |
| Early elder (65-74) | 1.18 (0.70, 1.99) |  |  |  |  |  | 0.90 (0.65, 1.22) |
| Late elder (≥75) | 0.67 (0.38, 1.17) |  |  |  |  |  | 0.61 (0.43, 0.87) ** |
| **Gender (Reference: Female)** |  |  |  |  |  |  |  |
| Male | 1.03 (0.78, 1.34) |  |  |  |  |  |  |
| **Presence of ≥ 1 comorbidity (Ref: No)** |  |  |  |  |  |  |  |
| Yes | 1.35 (1.02, 1.80)* |  | 0.83 (0.72, 0.96)* |  |  |  |  |
| **Type of TKA procedure (Ref: Bilateral TKA)** |  |  |  |  |  |  |  |
| Unilateral and staged TKA |  |  | 0.81 (0.57, 1.14) |  |  |  |  |
| **Surgical time, mins (Ref: ≤100)** |  |  |  |  |  |  |  |
| >100 |  |  | 0.94 (0.82, 1.08) |  |  |  | 1.00 (0.84, 1.20) |
| **Tourniquet time, mins (Ref: No or ≤100)** |  |  |  |  |  |  |  |
| >100 |  |  | 0.91 (0.79, 1.05) |  |  |  | 1.11 (0.92, 1.32) |
| **Type of implant model (Ref:** **Model J&J)** |  |  |  |  |  |  |  |
| Model S&N | 1.47 (0.98, 2.19) |  |  |  | 0.83 (0.68, 1.02) |  | 1.10 (0.84, 1.44) |
| Model Z | 1.48 (0.97, 2.26) |  |  |  | 0.87 (0.73, 1.04) |  | 0.79 (0.63, 1.00)* |
| Models S, B & Others | 0.90 (0.57, 1.43) |  |  |  | 0.78 (0.62, 0.98)* |  | 0.97 (0.74, 1.27) |
| **TP (Ref:** **Pharm & Mech)** |  |  |  |  |  |  |  |
| Pharm or Mech or None | 1.46 (1.02, 2.10)* |  |  |  |  |  | 1.11 (0.89, 1.37) |
| **Postoperative complications (Ref: No)** |  |  |  |  |  |  |  |
| Yes |  |  |  |  |  |  | 0.87 (0.70, 1.08) |
| **Length of stay, days (Ref: ≤ 5)** |  |  |  |  |  |  |  |
| **>** 5 |  |  |  |  |  |  | 1.11 (0.93, 1.32) |
| ***Adjustment variables*** |  |  |  |  |  |  |  |
| **Baseline physical function total** | 7.78 (5.92, 10.23) |  | 1.07 (0.98, 1.18) |  | 1.03 (0.93, 1.14) |  | 1.06 (0.93, 1.20) |
| **Year of surgery** | 0.99 (0.95, 1.04) |  | 1.00 (0.98, 1.01) |  | 1.02 (1.00, 1.04) |  | 1.23 (1.19, 1.27) |
| **Surgeon random effect variance** | 0.02 |  | 0.002 |  | 0.003 |  | <0.01 |
| **Model fit (Hat^2^ p-value)** | <0.001 |  | 0.86 |  | 0.72 |  | 0.15 |
| T_0_: Pre-op (baseline); T_1_: 6 months follow-up; T_2_: 1 year follow-up; T_3_: 2-year follow-up; T_4_: 5-year follow-up; N^a^: Total number of patients between two follow-up intervals; N^b^ /Total number of patients who showed improvement between two follow-up intervals; OR^c^ (95% CI): Odds ratio adjusted for baseline (T_0_) WOMAC score; TP: Thromboembolic prophylaxis; Pharm: Pharmacological thromboembolic prophylaxis that includes use of Low-Molecular-Weight-Heparins (LMWHs), heparins, and aspirin to prevent post-operative Venous Thromboembolism (VTE); Mech: Mechanical thromboembolic prophylaxis that includes compression devices such as thromboembolic deterrent stockings (TEDs), calf pumps and foot pumps to prevent post-operative VTE. **p*-value < 0.05; ***p*-value < 0.01. | | | | | | | |

## Table S5. Multivariable logistic analysis including all significant variables from univariate results (threshold P<0.1) accounting for year of surgery and baseline severity as fixed effect after multiple imputation with MICE algorithm

| **Variables** | **T_0_-T_1_** |  | **T_1_-T_2_** |  | **T_2_-T_3_** |  | **T_3_-T_4_** |
| --- | --- | --- | --- | --- | --- | --- | --- |
|  | **OR^c^ (95% CI)** |  | **OR^c^ (95% CI)** |  | **OR^c^ (95% CI)** |  | **OR^c^ (95% CI)** |
| **Total WOMAC score** |  |  |  |  |  |  |  |
| ***Variables of interests*** |  |  |  |  |  |  |  |
| Older adult (55-64) |  |  |  |  |  |  | 0.89 (0.66, 1.20) |
| Early elder (65-74) |  |  |  |  |  |  | 0.77 (0.58, 1.03) |
| Late elder (≥75) |  |  |  |  |  |  | 0.63 (0.46, 0.87)** |
| **Gender (Reference: Female)** |  |  |  |  |  |  |  |
| Male | 0.99 (0.73, 1.35) |  |  |  |  |  |  |
| **Presence of ≥ 1 comorbidity (Ref: No)** |  |  |  |  |  |  |  |
| Yes | 1.11 (0.80, 1.54) |  | 0.83 (0.71, 0.98)* |  |  |  |  |
| **Surgical time, mins (Ref: ≤100)** |  |  |  |  |  |  |  |
| >100 |  |  | 0.91 (0.80, 1.05) |  |  |  | 1.08 (0.92, 1.27) |
| **Tourniquet time, mins (Ref: No or ≤100)** |  |  |  |  |  |  |  |
| >100 |  |  | 0.92 (0.79, 1.06) |  |  |  | 1.18 (0.99, 1.42) |
| **Type of implant model (Ref:** **Model J&J)** |  |  |  |  |  |  |  |
| Model S&N |  |  |  |  |  |  | 1.04 (0.84, 1.28) |
| Model Z |  |  |  |  |  |  | 1.06 (0.82, 1.37) |
| Models S, B & Others |  |  |  |  |  |  | 0.85 (0.69, 1.05) |
| **TP (Ref:** **Pharm & Mech)** |  |  |  |  |  |  |  |
| Pharm or Mech or None | 1.41 (0.98, 2.02) |  |  |  |  |  | 0.87 (0.71, 1.08) |
| **Postoperative complications (Ref: No)** |  |  |  |  |  |  |  |
| Yes |  |  |  |  |  |  | 0.84 (0.69. 1.02) |
| **Length of stay, days (Ref: ≤ 5)** |  |  |  |  |  |  |  |
| > 5 |  |  | 1.11 (0.94, 1.30) |  |  |  | 0.98 (0.82, 1.16) |
| ***Adjustment variables*** |  |  |  |  |  |  |  |
| **Baseline WOMAC total** | 1.10 (1.09, 1.12) |  | 1.00 (1.00, 1.01) |  | 1.00 (0.99, 1.00) |  | 1.00 (0.99, 1.01) |
| **Year of surgery** | 1.01 (0.96, 1.05) |  | 0.99 (0.98, 1.01) |  | 1.00 (0.98, 1.02) |  | 1.02 (1.00, 1.05) |
| **Stiffness subscale score** |  |  |  |  |  |  |  |
| ***Variables of interests*** |  |  |  |  |  |  |  |
| **Gender (Reference: Female)** |  |  |  |  |  |  |  |
| Male | 1.18 (0.97, 1.43) |  | 0.88 (0.76, 1.02) |  |  |  |  |
| **Ethnicity (Ref: Chinese)** |  |  |  |  |  |  |  |
| Malay |  |  |  |  |  |  | 1.01 (0.79, 1.28) |
| Indian or others |  |  |  |  |  |  | 1.32 (1.05, 1.66)* |
| **Presence of ≥ 1 comorbidity (Ref: No)** |  |  |  |  |  |  |  |
| Yes | 0.90 (0.72, 1.11) |  |  |  |  |  |  |
| **Type of TKA procedure (Ref: Bilateral TKA)** |  |  |  |  |  |  |  |
| Unilateral and staged TKA |  |  |  |  |  |  | 0.61 (0.39, 0.94)* |
| **Blood loss, ml (s) (Ref: <250)** |  |  |  |  |  |  |  |
| ≥250 |  |  | 0.81 (0.65, 1.00)* |  |  |  | 0.69 (0.53, 0.89)** |
| **Type of implant model (Ref:** **Model J&J)** |  |  |  |  |  |  |  |
| Model S&N | 0.97 (0.72, 1.29) |  |  |  | 1.20 (0.97, 1.48) |  |  |
| Model Z | 1.06 (0.81, 1.39) |  |  |  | 0.95 (0.77, 1.16) |  |  |
| Models S, B & Others | 0.75 (0.54, 1.04) |  |  |  | 1.11 (0.88, 1.39) |  |  |
| **TP (Ref:** **Pharm & Mech)** |  |  |  |  |  |  |  |
| Pharm or Mech or None | 0.95 (0.74, 1.21) |  |  |  |  |  |  |
| ***Adjustment variables*** |  |  |  |  |  |  |  |
| **Baseline stiffness score** | 15.51 (13.35, 18.02) |  | 1.13 (1.06, 1.21) |  | 1.13 (1.05, 1.21) |  | 1.09 (1.01, 1.19) |
| **Year of surgery** | 0.93 (0.91, 0.96) |  | 1.00 (0.98, 1.02) |  | 1.03 (1.01, 1.05) |  | 1.11 (1.08, 1.14) |
| **Pain subscale score** |  |  |  |  |  |  |  |
| ***Variables of interests*** |  |  |  |  |  |  |  |
| **Gender (Reference: Female)** |  |  |  |  |  |  |  |
| Male | 0.93 (0.66, 1.31) |  |  |  | 0.93 (0.79, 1.09) |  |  |
| **Ethnicity (Ref: Chinese)** |  |  |  |  |  |  |  |
| Malay |  |  | 1.09 (0.90, 1.32) |  | 1.33 (0.73, 2.41) |  | 1.02 (0.83, 1.26) |
| Indian or others |  |  | 1.42 (1.17, 1.72)*** |  | 2.07 (1.25, 3.40)** |  | 1.38 (1.12, 1.69) ** |
| **BMI, kg/m^2^ (Ref: Obese (≥ 30))** |  |  |  |  |  |  |  |
| Underweight (<18.5) |  |  | 0.70 (0.29, 1.68) |  |  |  |  |
| Normal (18.5- 24.9) |  |  | 0.94 (0.80, 1.10) |  |  |  |  |
| Overweight (25-29.9) |  |  | 1.03 (0.88, 1.22) |  |  |  |  |
| **Presence of ≥ 1 comorbidity (Ref: No)** |  |  |  |  |  |  |  |
| Yes | 1.04 (0.72, 1.52) |  |  |  |  |  |  |
| **Type of anesthesia (Ref: GA)** |  |  |  |  |  |  |  |
| Non-GA |  |  |  |  |  |  | 1.26 (1.04, 1.53)* |
| **Blood loss, ml (s) (Ref: <250)** |  |  |  |  |  |  |  |
| ≥250 |  |  | 1.13 (0.92, 1.39) |  |  |  |  |
| **Type of implant model (Ref:** **Model J&J)** |  |  |  |  |  |  |  |
| Model S&N |  |  |  |  | 1.29 (1.01, 1.64)* |  |  |
| Model Z |  |  |  |  | 1.06 (0.86, 1.31) |  |  |
| Models S, B & Others |  |  |  |  | 1.11 (0.88, 1.41) |  |  |
| **TP (Ref:** **Pharm & Mech)** |  |  |  |  |  |  |  |
| Pharm or Mech or None |  |  | 0.92 (0.78, 1.08) |  |  |  |  |
| **Length of stay, days (Ref: ≤ 5)** |  |  |  |  |  |  |  |
| > 5 |  |  |  |  |  |  | 1.15 (0.95, 1.40) |
| **Ethnicity *Baseline pain score (Ref: Chinese)** |  |  |  |  |  |  |  |
| Malay |  |  |  |  | 0.85 (0.61, 1.18) |  |  |
| Indian or others |  |  |  |  | 0.79 (0.59, 1.06) |  |  |
| ***Adjustment variables*** |  |  |  |  |  |  |  |
| **Baseline pain score** | 31.74 (21.40, 47.10) |  | 1.20 (1.08, 1.33) |  | 1.23 (1.07, 1.42) |  | 1.10 (0.98, 1.23) |
| **Year of surgery** | 0.98 (0.94, 1.02) |  | 1.00 (0.98, 1.02) |  | 1.02 (1.00, 1.04) |  | 1.15 (1.12, 1.18) |
| **Physical function subscale score** |  |  |  |  |  |  |  |
| ***Variables of interests*** |  |  |  |  |  |  |  |
| **Age, years (Ref: Young adult (<55))** |  |  |  |  |  |  |  |
| Older adult (55-64) | 1.59 (0.93, 2.73) |  |  |  |  |  | 0.95 (0.71, 1.28) |
| Early elder (65-74) | 1.19 (0.70, 2.02) |  |  |  |  |  | 0.89 (0.67, 1.18) |
| Late elder (≥75) | 0.66 (0.38, 1.17) |  |  |  |  |  | 0.77 (0.57, 1.06) |
| **Gender (Reference: Female)** |  |  |  |  |  |  |  |
| Male | 1.06 (0.81, 1.39) |  |  |  |  |  |  |
| **Presence of ≥ 1 comorbidity (Ref: No)** |  |  |  |  |  |  |  |
| Yes | 1.29 (0.97, 1.72) |  | 0.84 (0.73, 0.98)* |  |  |  |  |
| **Type of TKA procedure (Ref: Bilateral TKA)** |  |  |  |  |  |  |  |
| Unilateral and staged TKA |  |  | 0.89 (0.63, 1.25) |  |  |  |  |
| **Surgical time, mins (Ref: ≤100)** |  |  |  |  |  |  |  |
| >100 |  |  | 0.93 (0.81, 1.06) |  |  |  | 1.05 (0.88, 1.24) |
| **Tourniquet time, mins (Ref: No or ≤100)** |  |  |  |  |  |  |  |
| >100 |  |  | 0.93 (0.80, 1.07) |  |  |  | 1.15 (0.94, 1.41) |
| **Type of implant model (Ref:** **Model J&J)** |  |  |  |  |  |  |  |
| Model S&N | 1.39 (0.95, 2.05) |  |  |  | 0.87 (0.72, 1.06) |  | 1.07 (0.85, 1.35) |
| Model Z | 1.33 (0.93, 1.91) |  |  |  | 0.87 (0.74, 1.03) |  | 0.92 (0.75, 1.13) |
| Models S, B & Others | 0.89 (0.57, 1.38) |  |  |  | 0.83 (0.68, 1.01) |  | 1.08 (0.85, 1.38) |
| **TP (Ref:** **Pharm & Mech)** |  |  |  |  |  |  |  |
| Pharm or Mech or None | 1.49 (1.06, 2.10)* |  |  |  |  |  | 0.97 (0.80, 1.19) |
| **Postoperative complications (Ref: No)** |  |  |  |  |  |  |  |
| Yes |  |  |  |  |  |  | 0.89 (0.73, 1.09) |
| **Length of stay, days (Ref: ≤ 5)** |  |  |  |  |  |  |  |
| **>** 5 |  |  |  |  |  |  | 1.05 (0.86, 1.28) |
| ***Adjustment variables*** |  |  |  |  |  |  |  |
| **Baseline physical function total** | 7.95 (6.05, 10.46) |  | 1.04 (0.95, 1.14) |  | 0.99 (0.90, 1.10) |  | 0.94 (0.82, 1.09) |
| **Year of surgery** | 1.01 (0.97, 1.05) |  | 0.99 (0.98, 1.01) |  | 1.01 (0.99, 1.03) |  | 1.10 (1.06, 1.13) |
| T_0_: Pre-op (baseline); T_1_: 6 months follow-up; T_2_: 1 year follow-up; T_3_: 2-year follow-up; T_4_: 5-year follow-up; N^a^: Total number of patients between two follow-up intervals; N^b^ /Total number of patients who showed improvement between two follow-up intervals; OR^c^ (95% CI): Odds ratio adjusted for baseline (T_0_) WOMAC score; TP: Thromboembolic prophylaxis; Pharm: Pharmacological thromboembolic prophylaxis that includes use of Low-Molecular-Weight-Heparins (LMWHs), heparins, and aspirin to prevent post-operative Venous Thromboembolism (VTE); Mech: Mechanical thromboembolic prophylaxis that includes compression devices such as thromboembolic deterrent stockings (TEDs), calf pumps and foot pumps to prevent post-operative VTE. **p*-value < 0.05; ***p*-value < 0.01. | | | | | | | |

## Table S6. Demographics of respondent vs non-respondents between consecutive follow-ups

| **Variables** | **T_0_-T_1_** | | **T_1_-T_2_** | | | **T_2_-T_3_** | | | **T_3_-T_4_** | | |
| --- | --- | --- | --- | --- | --- | --- | --- | --- | --- | --- | --- |
|  | **R, n (%)** | **NR, n (%)** | **R, n (%)** | | **NR, n (%)** | **R, n (%)** | | **NR, n (%)** | **R, n (%)** | | **NR, n (%)** |
| **Age, years** |  |  |  | |  |  | |  |  | |  |
| Young adult (<55) | 301 (6.4) | 15 (5.6) | 278 (6.3) | | 8 (4.3) | 266 (6.8) | | 12 (4.0) | 215 (7.8) | | 26 (3.1) |
| Older adult (55-64) | 1477 (31.5) | 94 (34.8) | 1389 (31.3) | | 60 (31.9) | 1250 (31.9) | | 91 (30.2) | 941 (34.2) | | 244 (28.9) |
| Early elder (65-74) | 2077 (44.2) | 106 (39.3) | 1981 (44.7) | | 76 (40.4) | 1754 (44.8) | | 124 (41.2) | 1174 (42.7) | | 360 (42.7) |
| Late elder (≥75) | 839 (17.9) | 55 (20.4) | 783 (17.7) | | 44 (23.4) | 649 (16.6) | | 74 (24.6) | 419 (15.2) | | 213 (25.3) |
| **Gender** |  |  |  | |  |  | |  |  | |  |
| Female | 3264 (69.5) | 184 (68.1) | 3079 (69.5) | | 128 (68.1) | 2740 (69.9) | | 202 (67.1) | 1961 (71.3) | | 564 (66.9) |
| Male | 1430 (30.5) | 86 (31.9) | 1352 (30.5) | | 60 (31.9) | 1179 (30.1) | | 99 (32.9) | 788 (28.7) | | 279 (33.1) |
| **Ethnicity** |  |  |  | |  |  | |  |  | |  |
| Chinese | 3361 (71.6) | 158 (58.5) | 3203 (72.3) | | 113 (60.1) | 2832 (72.3) | | 179 (59.5) | 2015 (73.3) | | 572 (67.9) |
| Malay | 705 (15.0) | 44 (16.3) | 660 (14.9) | | 27 (14.4) | 590 (15.1) | | 45 (15.0) | 387 (14.1) | | 112 (13.3) |
| Indian and others | 628 (13.4) | 68 (25.2) | 568 (12.8) | | 48 (25.5) | 497 (12.7) | | 77 (25.6) | 347 (12.6) | | 159 (18.9) |
| **BMI, kg/m^2^** |  |  |  | |  |  | |  |  | |  |
| Underweight (<18.5) | 37 (0.8) | 3 (1.1) | 34 (0.8) | | 3 (1.6) | 29 (0.7) | | 4 (1.3) | 22 (0.8) | | 9 (1.1) |
| Normal (18.5-24.9) | 1332 (28.4) | 73 (27.0) | 1259 (28.4) | | 60 (31.9) | 1118 (28.5) | | 92 (30.6) | 791 (28.8) | | 235 (27.9) |
| Overweight (25-29.9) | 1959 (41.7) | 95 (35.2) | 1855 (41.9) | | 64 (34.0) | 1634 (41.7) | | 115 (38.2) | 1137 (41.4) | | 346 (41.0) |
| Obese (≥ 30) | 1366 (29.1) | 99 (36.7) | 1283 (29.0) | | 61 (32.4) | 1138 (29.0) | | 90 (29.9) | 799 (29.1) | | 253 (30.0) |
| **Presence of ≥1 comorbidity** |  | |  | | |  | | |  | | |
| Yes | 3635 (77.4) | 200 (74.1) | 3443 (77.7) | 140 (74.5) | | 3018 (77.0) | 225 (74.8) | | 2102 (76.5) | 669 (79.4) | |
| No | 1059 (22.6) | 70 (25.9) | 988 (22.3) | 48 (25.5) | | 901 (23.0) | 76 (25.3) | | 647 (23.5) | 174 (20.6) | |
| **Type of TKA procedure** |  | |  | | |  | | |  | | |
| Bilateral TKA | 159 (3.4) | 24 (8.9) | 145 (3.3) | 18 (9.6) | | 127 (3.2) | 13 (4.3) | | 79 (2.9) | 35 (4.2) | |
| Unilateral and staged TKA | 4534 (96.6) | 246 (91.1) | 4285 (96.7) | 170 (90.4) | | 3791 (96.8) | 288 (95.7) | | 2669 (97.1) | 808 (95.8) | |
| **Surgical time, mins** |  | |  | | |  | | |  | | |
| ≤100 | 1855 (39.5) | 102 (37.8) | 1753 (39.6) | 76 (40.4) | | 1518 (38.8) | 122 (40.5) | | 1077 (39.2) | 364 (43.2) | |
| >100 | 2837 (60.5) | 168 (62.2) | 2676 (60.4) | 112 (59.6) | | 2399 (61.2) | 179 (59.5) | | 1671 (60.8) | 479 (56.8) | |
| **Tourniquet time, mins** |  | |  | | |  | | |  | | |
| No tourniquet | 46 (1.0) | 0 (0.0) | 44 (1.0) | 0 (0.0) | | 40 (1.0) | 1 (0.3) | | 30 (1.1) | 5 (0.6) | |
| ≤100 | 3061 (66.1) | 191 (73.2) | 2890 (66.1) | 133 (73.5) | | 2532 (65.2) | 203 (70.2) | | 1755 (64.4) | 585 (72.5) | |
| >100 | 1523 (32.9) | 70 (26.8) | 1438 (32.9) | 48 (26.5) | | 1311 (33.8) | 85 (29.4) | | 942 (34.5) | 217 (26.9) | |
| **Type of implant model** |  | |  | | |  | | |  | | |
| Model J&J | 920 (19.6) | 49 (18.2) | 870 (19.6) | 39 (20.7) | | 791 (20.2) | 61 (20.3) | | 573 (20.8) | 137 (16.3) | |
| Model S&N | 1134 (24.2) | 82 (30.4) | 1082 (24.4) | 52 (27.7) | | 887 (22.6) | 75 (24.9) | | 468 (17.0) | 284 (33.7) | |
| Models S, B & Others | 810 (17.3) | 47 (17.4) | 767 (17.3) | 28 (14.9) | | 714 (18.2) | 39 (13.0) | | 606 (22.0) | 101 (12.0) | |
| Model Z | 1828 (39.0) | 92 (34.1) | 1710 (38.6) | 69 (36.7) | | 1525 (38.9) | 126 (41.9) | | 1102 (40.1) | 321 (38.1) | |
| **Type of anesthesia** |  | |  | | |  | | |  | | |
| GA | 3752 (80.0) | 209 (77.4) | 3535 (79.8) | 148 (78.7) | | 3191 (81.5) | 241 (80.1) | | 2377 (86.5) | 605 (71.8) | |
| Non-GA | 939 (20.0) | 61 (22.6) | 893 (20.2) | 40 (21.3) | | 725 (18.5) | 60 (19.9) | | 372 (13.5) | 238 (28.2) | |
| **Blood loss, ml (s)** |  |  |  |  | |  |  | |  |  | |
| <250 | 3937 (88.9) | 222 (88.5) | 3712 (88.7) | 159 (86.9) | | 3465 (89.0) | 249 (88.3) | | 2408 (87.7) | 519 (88.1) | |
| ≥250 | 494 (11.2) | 29 (11.6) | 474 (11.3) | 24 (13.1) | | 430 (11.0) | 33 (11.7) | | 338 (12.3) | 70 (11.9) | |
| **TP** |  | |  | | |  | | |  | | |
| Pharm & Mech | 2757 (58.8) | 166 (61.5) | 2639 (59.6) | 112 (59.6) | | 2253 (57.5) | 172 (57.1) | | 1334 (48.5) | 585 (69.7) | |
| Pharm or Mech | 1868 (39.8) | 101 (37.4) | 1729 (39.1) | 73 (38.8) | | 1611 (41.1) | 124 (41.2) | | 1366 (49.7) | 246 (29.3) | |
| None | 63 (1.3) | 3 (1.1) | 57 (1.3) | 3 (1.6) | | 53 (1.4) | 5 (1.7) | | 49 (1.8) | 8 (1.0) | |
| **Post-op complication** |  | |  | | |  | | |  | | |
| No complication | 3707 (84.4) | 212 (85.8) | 3507 (84.4) | 151 (85.8) | | 3265 (84.3) | 227 (83.5) | | 2284 (83.8) | 483 (84.4) | |
| Complication | 687 (15.6) | 35 (14.2) | 647 (15.6) | 25 (14.2) | | 608 (15.7) | 45 (16.5) | | 441 (16.2) | 89 (15.6) | |
| **Length of stay, days** |  | |  | | |  | | |  | | |
| **≤** 5 | 3484 (74.5) | 203 (75.5) | 3292 (74.5) | 138 (73.4) | | 2867 (73.4) | 222 (73.8) | | 1886 (68.8) | 675 (80.3) | |
| **>** 5 | 1195 (25.5) | 66 (24.5) | 1125 (25.5) | 50 (26.6) | | 1039 (26.6) | 79 (26.2) | | 856 (31.2) | 166 (19.7) | |

T_0_: Pre-op (baseline). T_1_: 6 months follow-up. T_2_: 1 year follow-up. T_3_: 2-year follow-up. T_4_: 5-year follow-up; R: Respondents (patients who have <13 missing WOMAC items at consecutive follow-ups). NR: Non-respondents (patients who have > 13 missing WOMAC items at consecutive follow-ups); Others (Ethnicity): Minority populations that are not part of Singapore’s three main ethnic groups (Chinese, Malay, and Indians); TP: Thromboembolic prophylaxis; Pharm: Pharmacological thromboembolic prophylaxis that includes use of Low-Molecular-Weight-Heparins (LMWHs), heparins, and aspirin to prevent post-operative Venous thromboembolism (VTE); Mech: Mechanical thromboembolic prophylaxis that includes compression devices such as thromboembolic deterrent stockings (TEDs), calf pumps and foot pumps to prevent post-operative VTE.
